# A hybrid PKPD agent-based model of the tumour immune interaction: effects of anti-cancer combination therapy

**DOI:** 10.1007/s10928-026-10021-2

**Published:** 2026-03-24

**Authors:** Van Thuy Truong, Grant Lythe, Paolo Vicini, James W. T. Yates, Vincent F. S. Dubois

**Affiliations:** 1 Clinical Pharmacology and Quantitative Pharmacology, Clinical Pharmacology and Safety Sciences, AstraZeneca, Aaron Klug Building, Granta Park, Cambridge, CB21 6GH UK; 2 Department of Applied Mathematics, University of Leeds, Woodhouse Lane, Leeds, LS2 9JT United Kingdom; 3 Confo Therapeutics, Technologiepark 94, Ghent (Zwijnaarde), 9052 Belgium; 4 DMPK, Preclinical Sciences, RD Research, GSK, Gunnels Wood Road, Stevenage, Hertfordshire SG1 2NY United Kingdom

**Keywords:** Agent-based modelling, Cancer, Tutorial, Ordinary differential equation, Partial differential equation, Tumor microenvironment, Chemotherapy, Radiotherapy, DNA damage response inhibitor, PD1 antibody, Pharmacometrics, Pharmacokinetics, Pharmacodynamics, Quantitative systems pharmacology

## Abstract

We have developed a three dimensional hybrid multiscale agent-based ODE PDE model including tumour immune interaction, cell cycle phases, oxygen and drug diffusion dynamics, the pharmacodynamics of chemotherapy, targeted therapies, immunotherapy, radiotherapy and the respective systemic exposure levels of pharmacological treatments (described by pharmacokinetic modelling). The aim of this model is to support understanding of the spatial-temporal dynamic interactions between cancer cells, the relevant immune cells, and targeted therapies’ molecular moieties, which interact simultaneously in the tumour microenvironment. This interaction is further investigated in the context of combination therapies, thus making some inroads in the mechanistic understanding of positive and negative synergies when multiple therapies are administered. This work addresses the role of temporal sequencing in combination therapies, which requires the simultaneous modelling of multiple system components.

**Supplementary Information:**

The online version contains supplementary material available at 10.1007/s10928-026-10021-2.

## Introduction

Tumour survival and escape depend on various factors, including the immune response, concentration gradients (for example, those of oxygen and drug in the tumour micro environment (TME)), and on tumour cell characteristics (such as the location, cell-cycle phase and PDL1 expression status). In healthy tissue, the programmed cell death protein-1 (PD1) and its ligand PDL1 prevent excessive immune activity and serve as an immune checkpoint pathway to maintain ‘self’ tolerance. Under prolonged immune stress, PDL1 expression can be induced on cancer cells and other cells in the TME, producing an immunosuppressive environment [1]. Cell death due to radiotherapy can be induced by a combination of direct radiation-mediated cytotoxicity and the stimulation of an antitumor immune response, also known as the abscopal effect [2, 3]. Immune effector cells and suppressor cells can be subject to radiation-mediated cytotoxicity but more can be attracted by radiotherapy-induced cancer cell death [4]. In addition, radiotherapy-induced cancer cell death causes PDL1 overexpression [5]. Furthermore, the efficacy of radiotherapy depends on the oxygenation status of the tumour cells as hypoxic cells are less radiosensitive. Cell-cycle regulation also plays an important role. For example, cells in the G2-M phase are more sensitive to radiation than those in the G1 phase. Radiation can activate cell repair mechanisms such as the intracellular p53 and p21 pathways which results in cell cycle delay, accumulation in the G1 or G2 phase, and synchronisation [6]. DNA damage response inhibitors counteract these repair mechanisms by inhibiting ataxia telangiectasia mutated and rad3-related kinase [7]. Similarly to radiotherapy, the cell cycle plays a critical role in chemotherapy efficacy. For example, the drugs paclitaxel and docetaxel cause cells to accumulate at the G2-M phase, while flavopiridol treatment results in G1 and G2 phase accumulation [6, 8]. Additionally, chemotherapy causes higher immunogenicity by increasing the potential for cancer cell debris to be recognized by the immune system but can also lead to an immunosuppressive TME by overexpression of PDL1 on cancer cells and immune cell death [9, 10]. PDL1 or PD1 antibodies such as pembrolizumab block the interaction between PD1 and its ligands, PDL1 and PDL2. This blockade prevents effector cell exhaustion and facilitates tumuor killing and immune-mediated rejection [11]. Considering the characteristics of each treatment option, potential for significant combination benefit could be identified. For example, radiation-induced cell cycle delay can help various cell cycle phase-specific drugs to induce a higher cell kill, DNA damaging treatments such as radiotherapy or chemotherapy could be combined with DNA damage response inhibitors, and administration of PD1 antibodies can improve the immune response after radiation or chemotherapy treatment. Therefore, with the increasing complexity of mono and combination therapies, it is critical to understand those interactions, the heterogeneity of the tumour, the emergent behaviour in the TME, and the therapeutic effect of drug dose and schedule on a given tumour. Pharmacokinetic (PK) and pharmacodynamic (PD) models are used to predict dose and scheduling. However, those ordinary differential equation (ODE) models often require many states and an even larger number of parameters to capture the complex behaviour of many cell types. Additionally, spatial temporal dynamics, heterogeneity, emergent behaviour are often not incorporated into standard ODE PK-PD modelling approaches [12]. Therefore, we developed a multiscale hybrid agent-based partial differential equation (PDE) ODE model that incorporates tumour immune cell interaction which can be easily extended with different treatment modules such as radiotherapy, PD1 antibody, chemotherapy and DNA damage response inhibitor treatment.

## Methods

The hybrid multiscale immuno-oncology model consists of an agent-based model (ABM) describing the interaction between cancer cells and immune cells which is extended with ODEs and PDEs that simulate the oxygen distribution in the TME and different treatment modalities such as PD1 antibody treatment, radiotherapy, chemotherapy, and DNA damage response inhibitor.

### Agent-based model

**Fig. 1:**
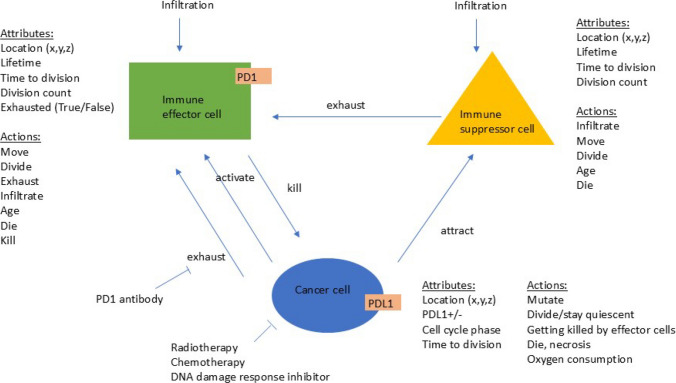
Schematic representation of the ABM. There are three types of agents: cancer cells, immune effector cells and immune suppressor cells. The attributes and actions of each type are shown. Cells are arranged on a three-dimensional grid

The ABM has three types of agents: cancer cells, immune effector and suppressor cells. Their attributes and actions are summarised in Fig. 1. We will refer to individual agents as cells; each has its *x*, *y* and *z* location on a 3D grid. The model does not include extracellular structures such as fibroblasts, vessels, or other cells. We scale to avoid long running times: one agent in the simulation represents $10^6$ biological cells. Thus, a 0.1cm$^3$ tumour that would contain about $10^8$ cells in reality is represented by 100 agents in the simulation [13]; a 1cm$^3$ tumour is 1000 cells in the simulation. The minimum detectable size in clinical conditions depends on various factors such as the tumour-to-background ratio, location of the tumour, imaging isotope and can range from 0.2-2cm$^3$ [14]. The lethal tumour size is 1000cm$^3$, represented by $10^6$ cells in the simulation [15, 16]. The immune cell to cancer cell ratio in the TME is 1:100 [17–22].

#### Cancer cells

PDL1 expression status, cell cycle phase, and time to division are attributes of cancer cells. They consume oxygen, mutate, age, divide, become quiescent, necrotic, and die due to natural death or due to therapy. A cancer cell ages and goes through the cell-cycle phases. After birth, a cell is in the G1 phase for 11h, then passes through subsequent phases as follows: S phase for 8h, G2 phase for 4h, and M phase for 1h [23]. Once a cancer cell is in the M phase, it is ready for division. After division, the cell cycle starts again [24]. Oncogenes can dysregulate the cell cycle and disturb the duration of the cell cycle phases and hence accelerate cancer cell population growth [25]; this is modelled by higher division rate in the Gillespie algorithm. When needed, the model rules for the duration of the cancer cell cycle can be changed for a cell or group of cells individually or for the whole population. This could be useful to simulate mutation of cells or a subgroup of cells that behaves differently to the rest of the population.

If the oxygen level is below a threshold or if there is no space next to a cancer cell for division, a cell enters the quiescent/resting phase (G0 phase) until the environmental condition changes. After reactivation, the cell goes back to the cell cycle stage where it was before going quiescent. (Oxygen levels can be increased and space can become available due to cells being eliminated by immune cell kill, radiotherapy, chemotherapy, DNA damage response inhibitor treatment, necrosis due to low oxygen levels, or natural death.) Division is implemented by selecting a cell which is ready for division randomly; the daughter cell is placed in one of the free positions next to the mother cell (in a 3D environment, there are 26 possible positions).

Cancer cells can become PDL1 positive due to genetic mechanisms such as CDK5 disruption, mutant EGFR, PTEN deletion, or PI3K/AKT mutations [26]. In addition to the constitutive PDL1 expression, interferon-inducible expression of PD-L1 is another pathway. The presence of tumor antigen–specific T cells that recognized the cancer cells leads to the production of interferon-gamma. This adaptive immune resistance appears in cancer tissue as a patchy pattern of PD-L1 expression. It is localized in T cell–rich regions of the tumor, particularly at the invasive margin [26]. To mimic those mechanisms, the simulation starts with PDL1-negative cancer cells, and in the course of the simulation, cancer cells can be randomly chosen to become PDL1-positive due to genetic events. Further, the PDL1 expression rate in the Gillespie algorithm increases with the number of cancer cells being eliminated by immune effector cells, simulating the adaptive immune resistance. To mimic that cancer cells in T cell-rich areas with high interferon-gamma gradient become PDL1 positive, the PDL1 status of the mother cancer cell is inherited to the daughter cell as the daughter cell is placed next to the mother cell in the simulation. The rates are implemented using the Gillespie algorithm. The oxygen levels in the environment are driving the cell behaviour. Cancer cells consume oxygen at a certain rate. Quiescent cells consume less oxygen than dividing cells.

#### Immune cells

Attributes of immune cells are: lifetime, time to division, division count, and exhaustion status for effector cells. Actions include infiltration into the TME, moving, dividing, aging, death, and (for effector cells) exhaustion and killing of cancer cells. In addition, immune suppressor cells can exhaust immune effector cells. Immune cells randomly infiltrate a free space in the environment. After infiltration, the division count, time to division, and age start at 0. While in the TME, immune cells can move to one, of the possible 26, adjacent locations. Immune effector cells are attracted by cancer cells, so they move with a higher likelihood to a location which is closer to a cancer cell. Immune suppressor cells have the goal of exhausting immune effector cells and move with a higher likelihood closer to an effector cell position. Exhaustion happens with a certain rate simulated with the Gillespie algorithm when an immune effector cell is next to a PDL1+ cancer cell or an immune suppressor cell. After exhaustion, the effector cell becomes inactive and is not able to move, kill, or divide. The exhausted cells are cleared from the environment once the lifespan is reached. Effector cells can kill cancer cells when they are next to a cancer cell. Immune cells age with the time in the simulation. They die and are cleared from the system when their age is above the lifespan or due to cell death because of radiation or chemotherapy. Immune cell division happens at a certain rate when the time to division is reached and the division count is not exhausted. The daughter cell is placed randomly in one of the free locations next to the mother cell. The division count and time to division of the mother cell is updated after the division event; the daughter cell inherits the division count, and starts the time to division, and lifespan at 0 [17].

### Gillespie algorithm and rates

The actions are chosen according to the Gillespie algorithm [12, 27] and their probabilities are set by rates multiplied by certain cell numbers. Cancer cell division happens with the division rate times the number of existing cancer cells in the environment which makes division more likely the higher the cancer cells numbers are. A PDL1 expression event is more likely when more cancer cells are being eliminated by effector cells, chemotherapy or radiotherapy. The cancer kill probability increases with the number of effector cells while the natural cancer cell death rate depends on the number of cancer cells. The immune effector infiltration rate increases with the number of cancer cells, immune effector cells in the TME and eliminated cancer cells by the immune reaction while the suppressor infiltration rate depends on the number of tumour cells that have been killed by effector cells. Immune cell division and moving rates are higher with a larger number of immune cells. The effector exhaustion rate depends on the number of suppressor cells, PDL1+ cancer cells and the receptor occupancy with the antibody. Those rates and their dependencies can be changed to account for different tumour types. More details about the parameter values can be found in the supplementary material.

#### Oxygen in the environment

The oxygen diffuses from the periphery to the tumour as in our examples no blood vessels are simulated. The entry point for oxygen can be set on specific points on the grid to model blood vessels. Actively dividing cancer cells consume oxygen with the consumption rate $k_o$ while the oxygen consumption of quiescent cells is reduced by the factor $q_c$. The parameters are assumed and can be changed to simulate different degrees of oxygenation in the tumour micro environment and oxygen consumption rates of the cancer cells. We set the initial condition of oxygen as 1 and measure the concentration of oxygen in TME relative to it initial condition. Cancer cells go into the division, quiescent, and necrotic stage according to the available oxygen level in the TME. The work of Salem et al. [28] can be used to calibrate the cancer cell behaviour in the tumour microenvironment depending on the oxygen gradient. The PDE equation and parameter table can be found in the supplementary.

### Treatment

The PD1 antibody treatment module is implemented with a PKPD model from the literature [11] where a system of ODEs is used to calculate the receptor occupancy of pembrolizumab on the PD1 effector cell receptor which will determine the effector cell exhaustion rate.

Radiation has a twofold effect. Due to the cytotoxicity caused by irradiation, the immune cells as well as the cancer cells die and as a consequence generate immunogenic tumour debris. This immunostimulatory effect mediated by immunogenic cell death provokes immune cell infiltration after an initial immune suppression by radiation and hence a delayed antitumour immune response [29]. In the radiotherapy module, the survival probability of each cell after radiation is simulated with a modified linear-quadratic model and oxygen modification factor according to Powathil et al. [6]. The cell cycle delay of cancer cells in the G1 and G2 phase after radiation is drawn from a uniform distribution between 1-9h [6]. A cell survives if a random number drawn from a uniform distribution between 0-1 is smaller than the modified survival probability and dies otherwise. The delayed immune cell infiltration after radiation is modelled deterministically according to Alfonso et al. [4] which depends on the number of administered radiotherapy doses time of irradiation, and number of eliminated cancer cells. More information can be found in the supplementary.

The drug therapies are similated with an ODE based model. To take into consideration that the drug distribution differs throughout the 3D lattice, the drug concentration is scaled based on the predicted oxygen gradient. Similarly to the oxygen supply, the drug diffuses from the periphery as no blood vessels are simulated. This simulates the lower concentration of drug in the tumour core in comparison to the tumour edge.

For the chemotherapy module, we simulated treatment with docetaxel with a K/PD model from Frances et al. [30]. Chemotherapy causes a kill rate which depends on the time, the amount of drug in the system, the efficacy rate, and the resistance parameter. The resistance parameter causes an exponential decline in the kill rate, hence making the chemotherapy less effective over time (see equation 19 supplementary information). The chemotherapy will affect immune cells and cancer cells in the G2 and M phases [8]. The death probability is calculated by the kill rate divided by the maximum kill rate. A random number is drawn from a uniform distribution, if it is smaller than the survival probability, the cell with survive and die otherwise.

The DNA damage response inhibitor treatment module is based on a PKPD model from Terranova et al. [31]. The drug effect at time *t* and at a certain location is modelled by an Emax model. The drug only affects cancer cells in the S phase. A random number is drawn and the cell dies if that number is bigger than the survival probability. More details can be found in the supplementary material.

## Results

The sensitivity analysis (see supplementary material) has revealed which parameters to change in order to change the tumour immune dynamics and the immune exhaustion pathway in order to decrease the tumour growth and eliminate the cancer population. In this section, we simulate different tumours with their parameters with and without treatment to find out how certain treatment regimes affect the tumour immune interaction.

### Tumour immune cell interaction without treatment

**Fig. 2:**
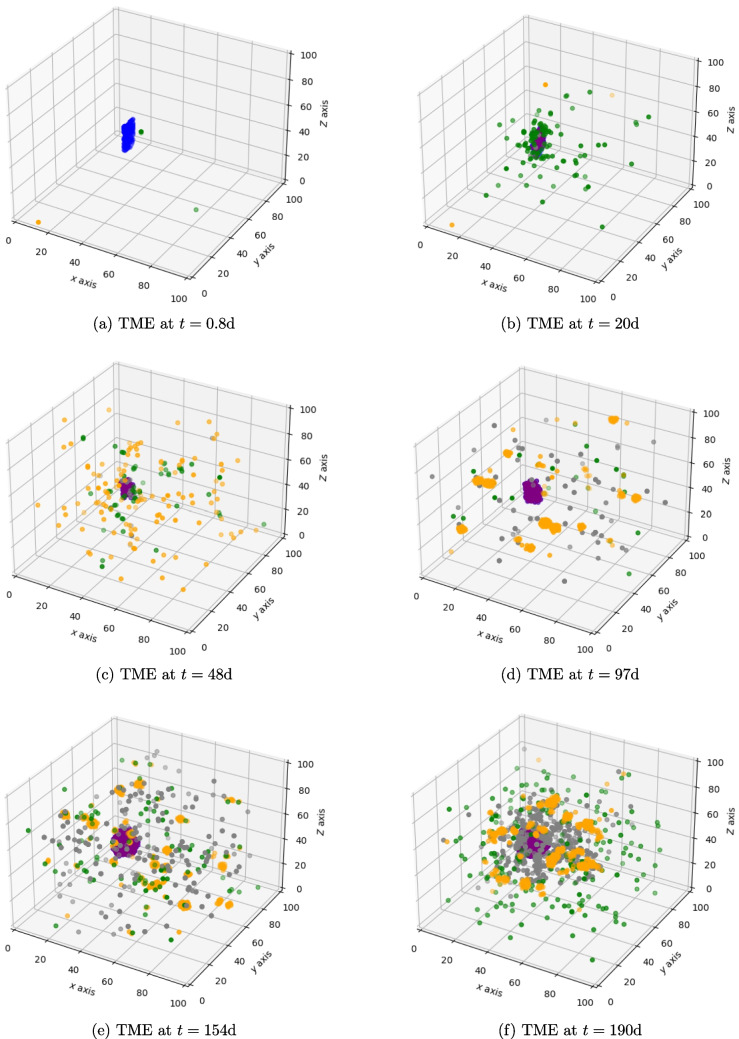
Figures 2a–f show the interaction of tumour and immune cells over time

**Fig. 3:**
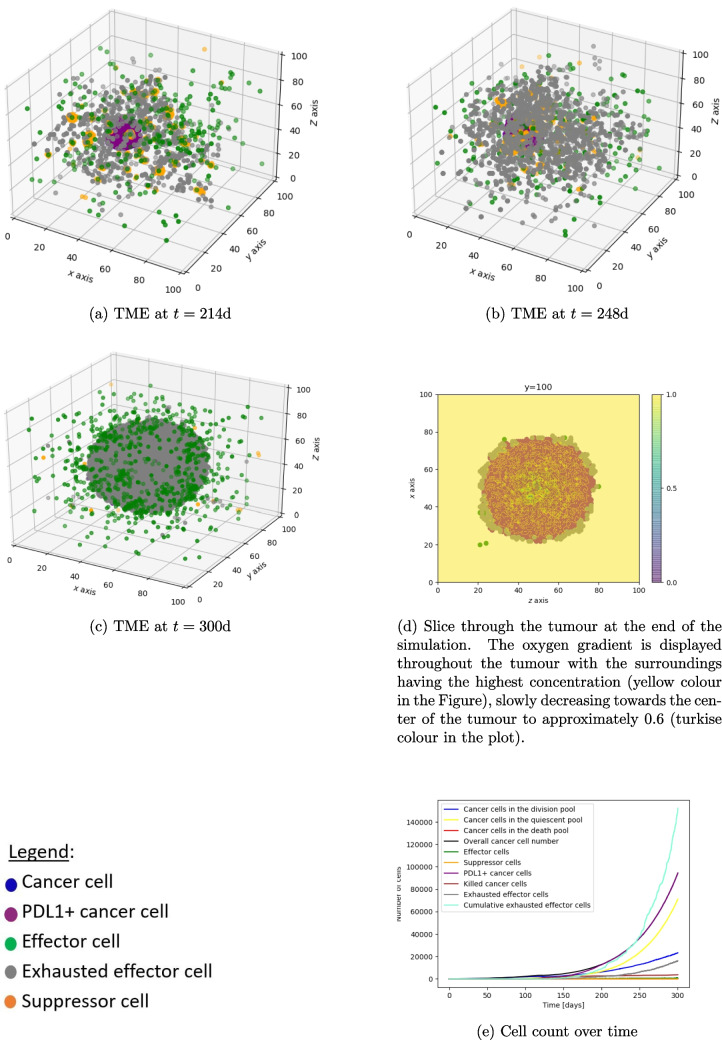
Figures 3a – d show the interaction of the cancer cells with the immune system over time and at the end of the simulation. Figure 3e shows the cell count over time. A movie can be found here https://github.com/VanThuyTruong/tumour_immune_abm

We see, in Fig. 2a-b, that when simulating a tumour starting with 100 cancer cells without treatment intervention, the cancer grows and attracts immune cells. Effector cells eliminate some cancer cells and, as a result, the cancer will express PDL1. Interactions between immune effector cells and PDL1+ cancer cells and infiltrated suppressor cells cause effector cell exhaustion (Fig. 2c-f). Over time the TME becomes more and more immunosuppressive (Fig. 2c-f and Fig. 3a-c). At the end of simulation (300 days), a front is formed between the immune cells and the cancer cell population with PDL1 positive cancer cells surrounded by exhausted PD1 positive effector cells clustering at the interface (Fig. 3c-d) [32–35]. Overall, the cancer cell population continues to grow as the oxygen consumption has not yet caused hypoxia (Fig. 3e).

### PD1 antibody treatment

**Fig. 4:**
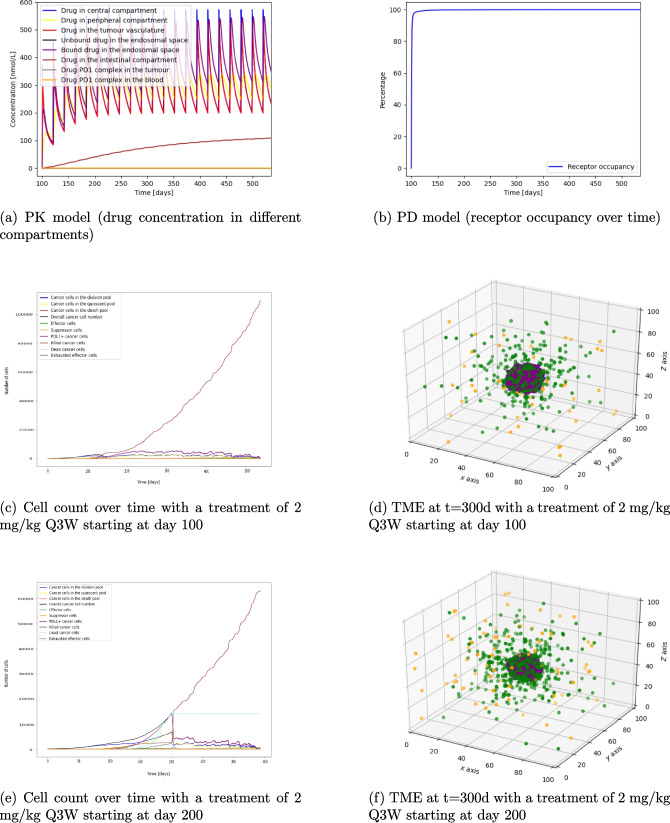
Figures 4a – f show the PKPD of a PD1 antibody treatment and the interaction of tumour and immune cells over time with different treatment schedules

A PD1 antibody treatment with a dose of 2 mg/kg Q3W is simulated. The pharmacokinetics of the antibody, including the drug distribution in the central and peripheral compartment, tumour vasculature, endosomal space, intestinal compartment and the drug PD1 complex, can be seen in Fig. 4a. This treatment schedule leads to almost 100% receptor occupancy (see Fig. 4b) which prevents immune effector cell exhaustion. To show the importance of the immune cell infiltration, a tumour parameter set with a slow immune cell infiltration is chosen. Starting treatment at day 100 with 105 infiltrated effector cells and 1.5% PDL1 mutated cancer cells, the cancer cell growth decreases by 51% in the first two months and tumour extinction happens at day 535 (Fig. 4c). Starting at day 200 with the same treatment and 100% mutated cancer cells, 1400 exhausted effector cells, and 310 effector cells, extinction happens at day 345 (Fig. 4e). This simulation shows the importance of immune effector cell infiltration. The treatment is more effective when more immune effector cells are in the TME at that time which can contribute to cancer cell elimination. Figure 4d and f, which are snapshots of the TME taken at the same time point, but with different treatment start times, emphasize this. In the case of early PD1 treatment (Fig. 4d) less immune effector cells have infiltrated the TME. Therefore, even though immune cell exhaustion does not happen since the PD1 receptor is blocked before the majority of cancer cells become PDL1 positive, the time to tumour extinction is longer (after 535 days) in comparison to the case of a later treatment starting at 200 days (extinction happens at day 345). In addition, by keeping the tumour small less tumour antigen is produced and therefore less immune effector cells are being attracted into the tumour micro environment. This shows that for the PD1 antibody treatment it is important to test for sufficient immune effector cell infiltration and PDL1 mutation status of the tumour before treatment. Without sufficient immune effector cell infiltration combination therapy or other treatment methods could be considered.

### Radiotherapy

**Fig. 5:**
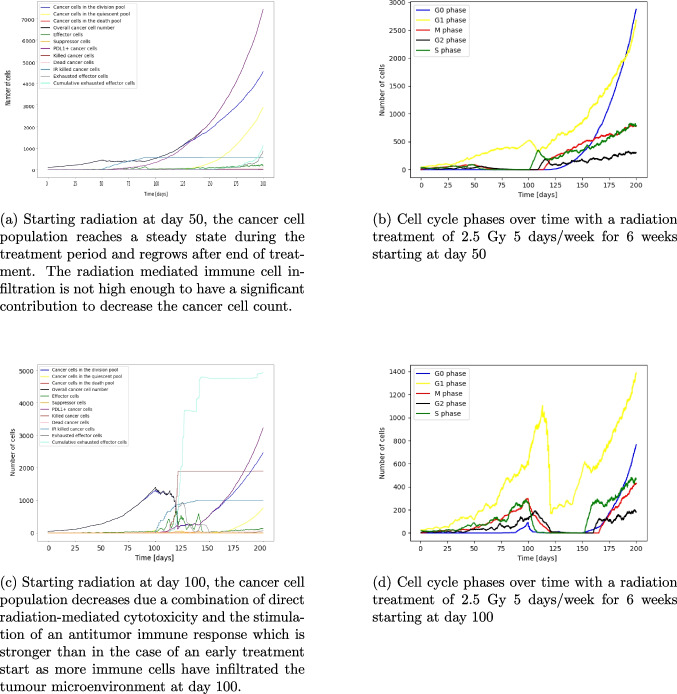
Figures 5a – d show the effect of radiotherapy with a weekly dose of 2.5 Gy/day for 5 days/week for 6 weeks starting at day 50 and day 100

Investigating radiotherapy also shows the importance of the immune reaction. The radiation causes cancer cell death and creates debris which increases the immune effector cell infiltration. Radiotherapy is given as a treatment of 2.5 Gy 5 days a week for 7 weeks [36–38]. Starting at day 50, less immune effector cells have infiltrated the TME. Therefore, even with the increased immune response after radiation the cancer cell population reaches a steady state during treatment and growth is continuing after end of treatment (see Fig. 5a). In comparison, at day 100 more immune effector cells are in the TME. The combination of radiotherapy induced cytotoxicity and immune cell infiltration decreases the cancer cell count by around 96% at the end of treatment (Fig. 5c). Due to radiation and cancer cell elimination by immune cells, PDL1 positive mutants emerge and cause effector cell exhaustion. Despite this decrease the tumour population can recover and increases exponential after the treatment finishes. Those simulations also show that radiotherapy affects the cell cycle phases differently and causes cell cycle delays and redistribution (see Fig. 5b, d). For example, in Fig. 5d it can be seen that at day 100 before the treatment starts, there are around 100 cells in the G0 phase, around 150 cells in the G2 phase, around 200 cells each in the S and M phase and around 600 cells in the G1 phase. At day 110, ten days into treatment, the number of cells in the G0 phase is close to zero as those cells are becoming active again after cells around them die and make space for quiescent cells being reactivated. The number of cells in the G2 phase is higher than the number of cells in the S and M phase (about 180 cells in the G2 phase, 150 cells in the M phase and around 10 cells in the S phase). Before treatment start, it was the opposite. This is because the cell cycle delay happens in the G2 and G1 phase due to radiotherapy. Hence more cells are stuck in those phases. Additionally, to the cell cycle delay cells in the G1 phase are less sensitive to the radiation than cells in the S, M and G2 phase. Even though the sensitivity for the S, M and G2 phase is the same, more cells are in the M phase than S phase during radiotherapy as there are more cells in the G2 phase which can go into the next phase, the M phase. At around day 120, the number of cells in the G1 phase drops as the number of cells in the M phase decreases close to 20 cells, hence less cells can cycle from the M to the G1 phase. After the end of treatment at day 149, the cancer cells slowly recover. Unlike before treatment there are now more cells in the S phase than in the M phase (at day 160 around 300 cells in the S phase and around 10 in the M phase). This is caused by the efflux of cells from the G1 phase into the S phase. The G2 phase recovers before the M phase, as S phase cells transition into the G2 phase. The cell cycle delay is a resistance mechanism enabling the cancer cell to remain longer in certain cell cycles to activate pathways to repair the double and single DNA strand break caused by radiation [39].

**Fig. 6:**
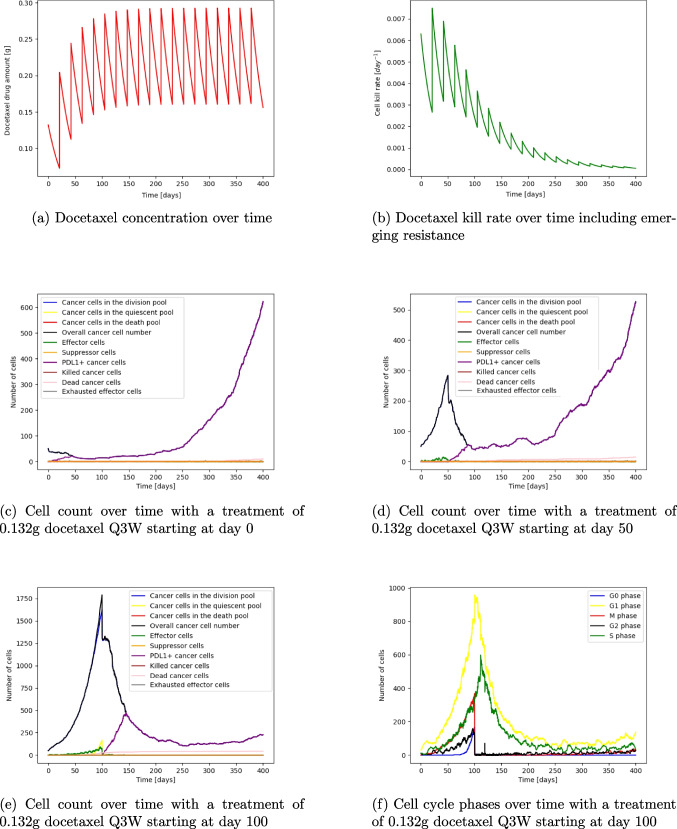
Figures 6a – f show the chemotherapy treatment with 0.132g docetaxel Q3W with the resulting drug concentration, kill rate, cell count and cell cycle phases

### Chemotherapy

Looking into chemotherapy with docetaxel 0.132g Q3W (based on a patient body surface area of 1.75 $m^2$ and a 75 $mg/m^2$ dose [30]) we can see that chemotherapy has different impact depending on the tumour size. The administration of 0.132g docetaxel Q3W with its concentration over time is shown in Fig. 6a. This causes a kill rate which decreases over time due to the emerging resistance of the cells (Fig. 6b). Starting the treatment at day 0 initially decreases the cell count, and causes a plateau in the cancer cell count until resistance emerges and the tumour can grow exponentially to around 600 cells at the end of the simulation (Fig. 6c). Starting treatment at day 50, a sharp decrease after the initial dose emerges as docetaxel cause cell death of cancer cells in the drug-sensitive G2 and M phase (from approximately 290 cells to 200 cells). The cancer cell number decreases steadily to around 50 cells until resistance emerges around day 220 and the tumour grows again until over 500 cells at the end of the simulation (Fig. 6d). The same treatment beginning at day 100 has a different impact on the cancer cell dynamics. Initially, there is a sharp decline in the cell number after the first dose (approximately 1800 cells to 1250 cells) which follows a time span of approximately 20 days with an almost constant cell number of approximately 1250 cells and then a sharp decline again to approximately 200 cells around day 200 (Fig. 6e). Looking at the plots with the cell cycle phases over time the reason for this plateau can be seen. After the initial cell kill of cells in the G2 and M phase, the cells in the dormant state become active again as the cell death frees up space and oxygen supply can increase. Dormant cells can enter the cell cycle and increase the cells in the G2 and M phases which are susceptible to chemotherapy (Fig. 6f). Eventually, the cancer cells will become resistant to the treatment and from around day 300 the cancer cell number slowly rises again. This shows that chemotherapy can make dormant cells active again which might not be sensitive to drugs in their inactive state.

### DNA damage response inhibitor

**Fig. 7:**
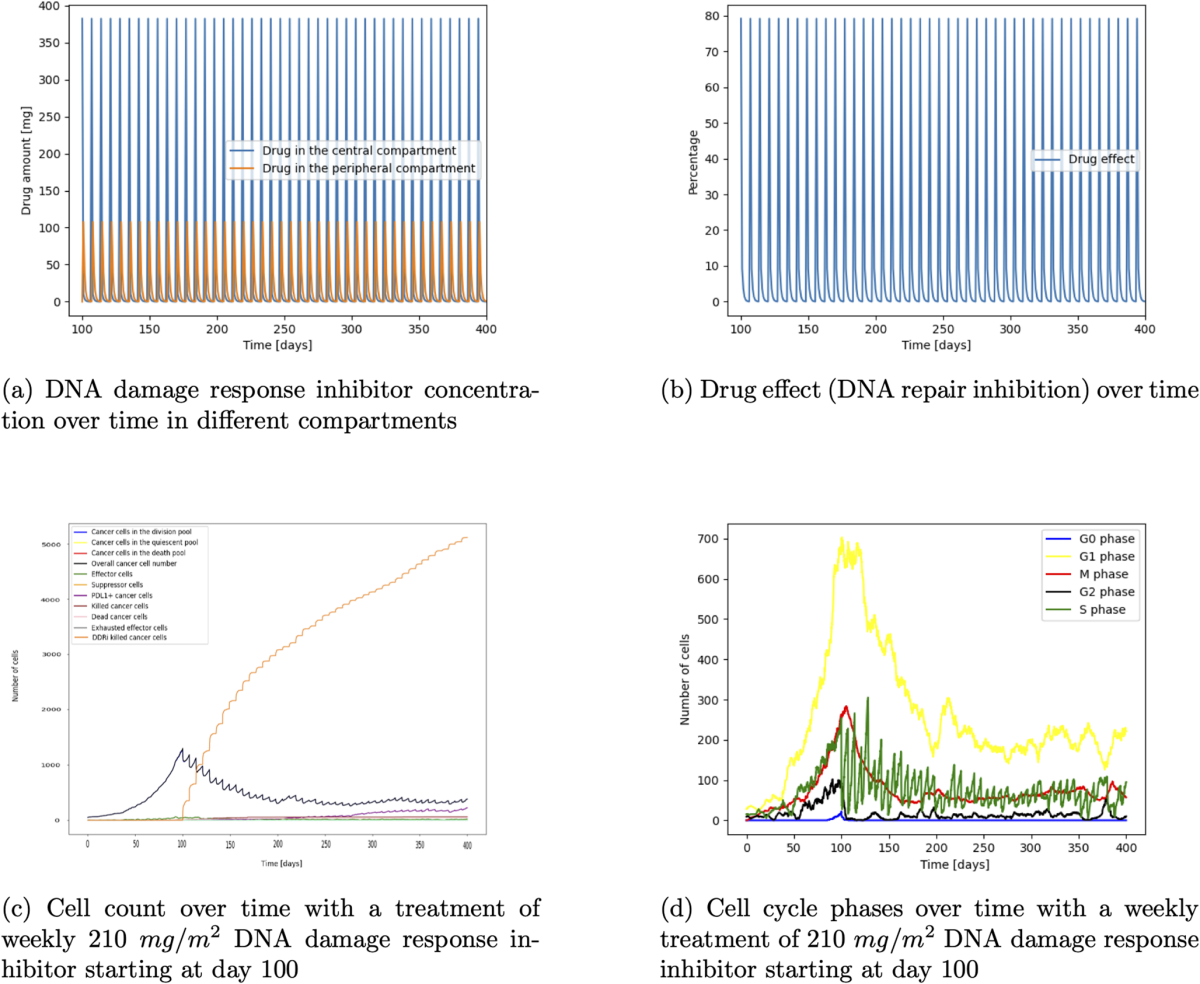
Figures 7a – 8d show the DNA damage response inhibitor treatment with weekly 210 $mg/m^2$ with the resulting pharmacokinetic and pharmacodynamic, cell count, rates of cell interaction, and the TME

Investigating the DNA damage response inhibitor treatment we can see the drug concentration in different compartments from a weekly dose administration of 210 $mg/m^2$ (Fig. 7a) and the resulting DNA repair inhibition (Fig. 7b). Figure 7c shows that treatment starting at day 100 causes a decline in the cell count from around 1400 cells to a steady state of around 500 cells. We can see in Fig. 7d that after treatment starts at day 100, the cell number in the S phase oscillates as the treatment affects this cell cycle phase. After treatment start at day 100, the cell number drops from approximately 250 cells to around 30 cells. Cells from the G1 phase enter the S phase and due to the weekly administration, the the cells in the S phase can recover which causes an increase to approximately 230 cells at day 107. Then the next dose is given which decreases the cells in the S phase to approximately 20 cells after day 107. This oscillation continuous to decrease the overall cell number until around day 200 when the cell number reaches a steady state and the S phase cells oscillate between approximately 130 cells to 20 cells.

**Fig. 8:**
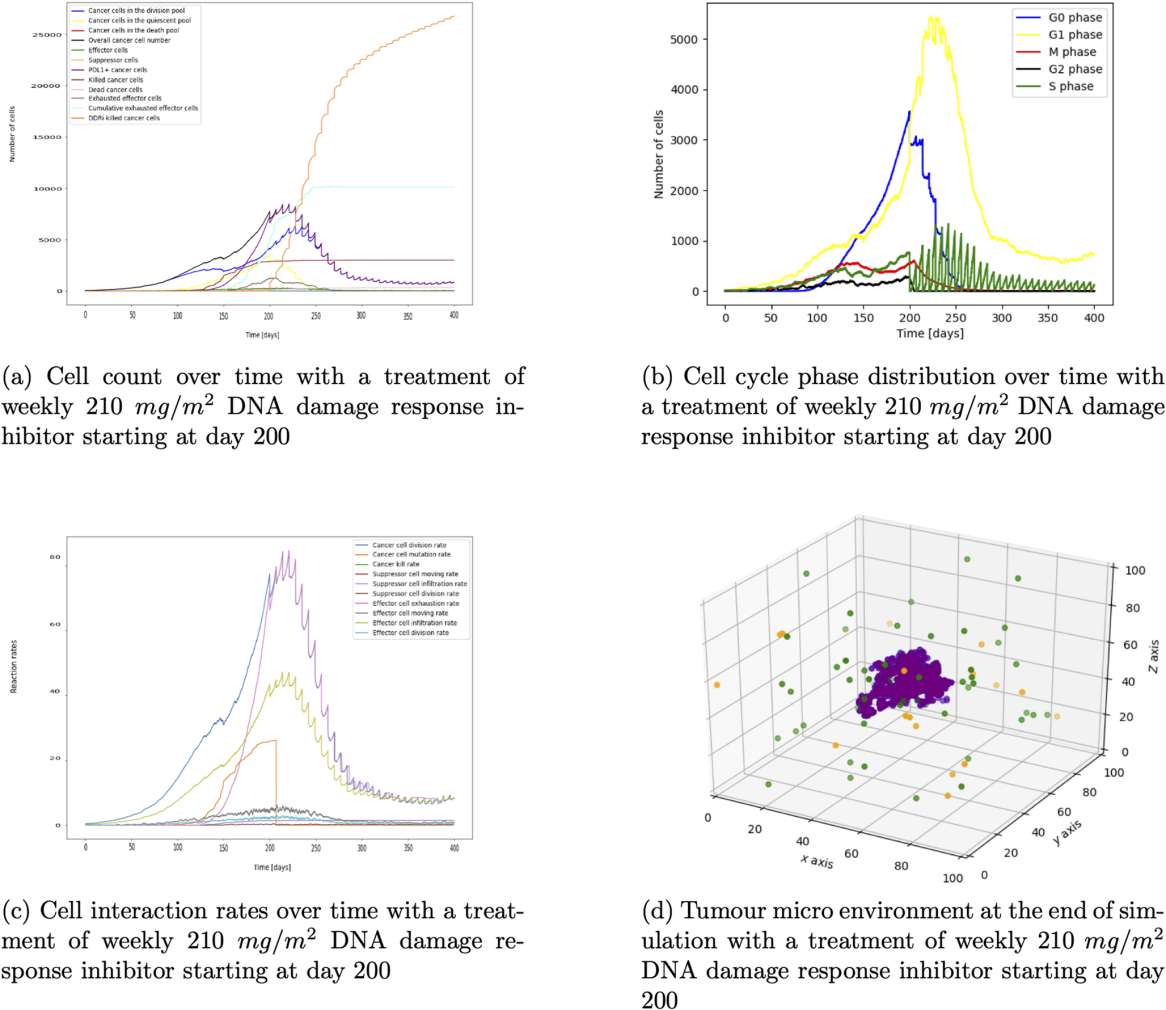
Figures 7a – 8d show the DNA damage response inhibitor treatment with weekly 210 $mg/m^2$ with the resulting pharmacokinetic and pharmacodynamic, cell count, rates of cell interaction, and the TME

Figure 8a displays not only a decline in cancer cell numbers after the start of treatment at day 200 but also shows a decrease in exhausted effector cells. The plot with the rates (Fig. 8c) emphasizes this by showing that effector cell exhaustion decreases after the start of treatment on day 200. This is because the effector exhaustion rate in the Gillespie algorithm depends on the number of PDL1+ cancer cells which decrease due to the DNA damage response inhibitor treatment. The cancer cell count reaches a steady state of around 500 cells. Looking at Figs. 7c and 8a we can see that the DNA damage response inhibitor treatment only suppresses the cancer growth but does not eliminate the tumour because it only lowers the exhaustion rate but does not increase immune effector cell infiltration (see Fig. 8c for the rates). Comparing the plot of the TME (Fig. 8d) with the TME during PD1 antibody treatment (Fig. 4d and f) we can see that there is not a sufficient number of immune effector cells to eliminate the cancer (For an easier comparison the plots with the rates and tumour microenvironment are being displayed next to each other in Figures S12 and S13 in the supplementary).

**Fig. 9:**
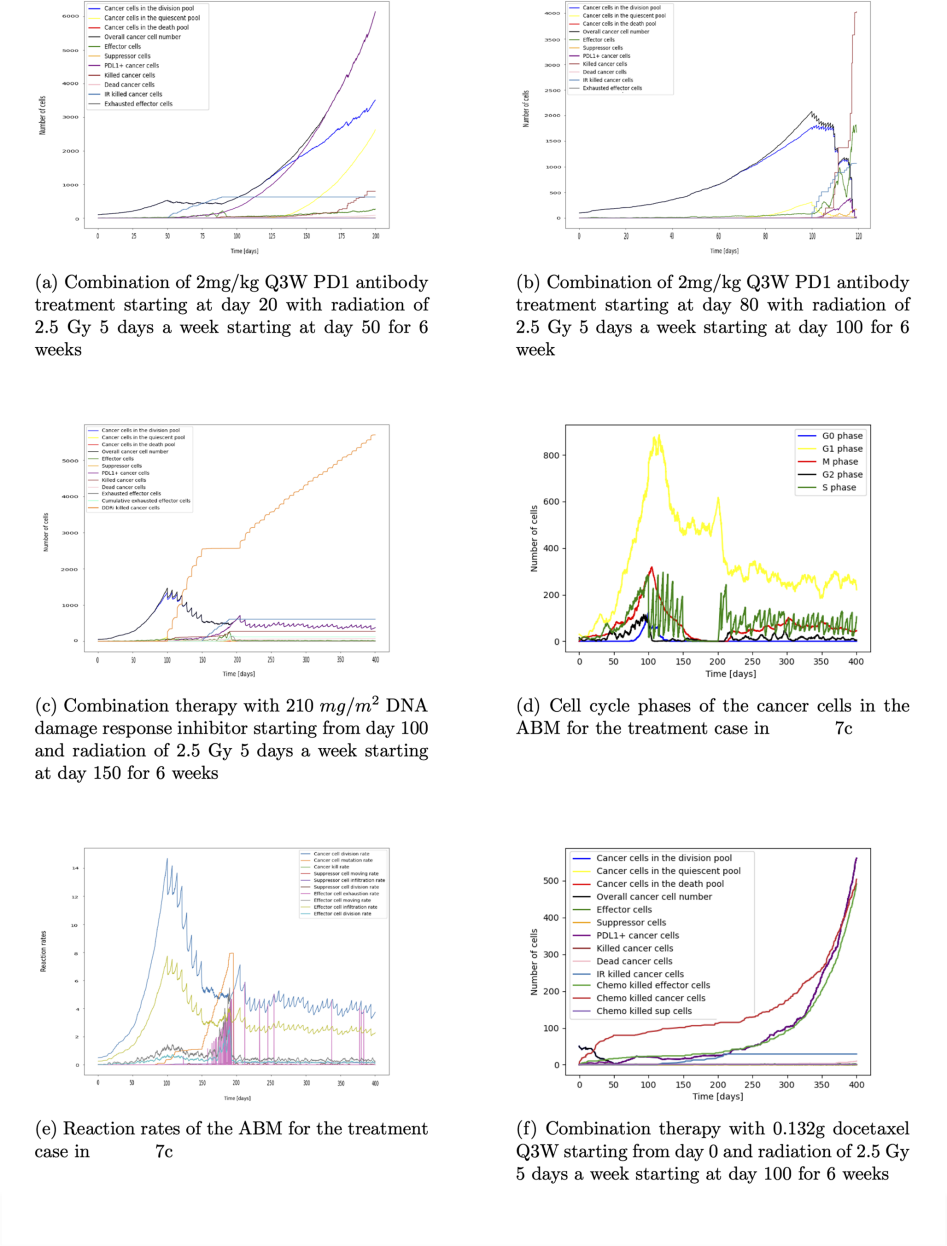
Figures 9a – f show different options for combination treatment and their impact on the cell count and cell interaction rates over time

### Combination therapy

The previous analysis has shown that most of the single compound treatment can decrease the cancer cell count but not eliminate the whole tumour. Hence, we now simulate combination treatment to understand interactions between different treatment options and the interplay with the immune system to see which combinations can overcome the exhaustion mechanism and eliminate the cancer population.

Looking at the combination of PD1 antibody with radiotherapy, we see the involvement of the immune system is important. To introduce the subsequent analysis, the PD1 antibody treatment is given prior to radiotherapy as a multiple dose regime which will continue until the end of simulation. In Fig. 9a we can see that the outcome of the combination therapy with 2 mg/kg PD1 antibody Q3W starting at day 20 with radiation of 2.5 Gy 5 d/week starting at day 50 for 6 weeks is similar to the case of radiation mono therapy with the same schedule (see Fig. 5a for comparison or Figure S13 in the supplementary with both treatment plots next to each other). In contrast, combining PD1 antibody with a later radiation treatment starting at day 100 causes tumour extinction (see Fig. 9b). This is caused by the higher immune cell infiltration at the later time. In addition, radiotherapy causes debris. This acts as an in situ vaccine which further attracts immune effector cells to the tumour micro environment [29, 40]. Therefore, the cancer population extinction is caused by a combined effect of radio toxicity, immune cell infiltration and immunogenetic cancer cell elimination. This example shows that an established immune response to cancer is crucial to aid treatment success. At the later time point more immune cells have infiltrated and additionally, the PD1 antibody is given to prevent immune cell exhaustion. Hence the immune system can work more efficient in eliminating the cancer cells in addition to the radiotherapy. More information about adapting radiotherapy to the immune response can be found in the review article of Galluzzi et al. [41]. This shows that a high immune cell count aids treatment success.

The combination of a DNA damage response inhibitor with radiotherapy demonstrates the importance of finding the optimal schedule. The simulation shows that treatment with weekly 210 $mg/m^2$ DNA damage response inhibitor starting from day 100 until the end of simulation and 2.5 Gy radiation for 5 days a week starting from day 150 for 6 weeks is detrimental. When starting the DNA damage response inhibitor early at day 100, the immune response has not been established and only a low number of immune effector cells have infiltrated the TME (Fig. 9c). Decreasing the cancer cell count with the DNA damage response inhibitor lowers the antigenicity and immune cell infiltration as in this model the immune effector cell infiltration rate depends on the number of cancer cells in the TME. When the radiation starts at day 150, immune cells and cancer cells are eliminated due to radio-toxicity. Radiation-induced cancer cell death leads to an increase in PDL1 expression. This causes a higher immune effector cell exhaustion rate (see Fig. 9e). In combination with the decreased immune effector cell count and the increased immune cell exhaustion, the equilibrium of cancer growth and elimination by the immune system is disturbed and the tumour can grow. This causes a peak in the number of exhausted effector cells around 190 days and a peak in the cancer cell count around day 210. The increased immune cell infiltration due to the cancer cell debris caused by radiotherapy between day 150 and 200 decreases the cancer cell count after the peak around day 210 and leads to an equilibrium again from approximately day 225. Figure 9d shows the impact of treatment on the cell cycle phases. DNA damage response inhibitor treatment decreases the cells in the S phase in an oscillating manner as it is given weekly. Between 150-200 days, radiotherapy decreases the cell count in all phases. Swapping the sequence of radiotherapy and DNA damage response inhibitor treatment (see Figure S14 in the supplementary) shows a similar effect with the DNA damage response inhibitor eliminating the cancer cells in the S phase and the radiotherapy increasing the number of immune effector cells but also the PDL1 positive cancer cell mutation and immune effector cell exhaustion.

Looking at the combination of 0.132g Q3W docetaxel treatment starting at day 0 with 2.5 Gy 5 days a week radiation starting at day 100 for 6 weeks, we can see that the effect of radiation is negligible. Until the end of radiation, approximately 50 cancer cells have died due to the radiotherapy (Fig. 9f). Once resistance to the chemotherapy emerges the cancer grows exponentially to around 600 cells at the end of the simulation. In contrast, radiation alone with the same administration schedule is able to decrease the cancer cell number by around 96% (see Fig. 5c). This example demonstrates the important contribution of the immune system to tumour extinction. Because ongoing chemotherapy has eliminated immune cells, radiation is less effective. While methodologically interesting, these results would of course require independent experimental validation for confirmation.

## Discussion

Our three-dimensional multiscale hybrid ABM PDE ODE model incorporates tumour-immune cell interaction and different treatment modules: radiotherapy, PD1 antibody, chemotherapy and DNA damage response inhibitor treatment. For chosen parameter sets, we have simulated the trajectories of tumours without therapy and with monotherapy or combination therapy.

The agent-based framework has the advantage of providing spatial interaction and being a rule-based stochastic bottom up method. Heterogeneity such as different spatial location of the cancer cells, mutation rates, susceptibility to treatment can be easily simulated. Therefore, emerging behaviour from the interplay between the different cell types and the consequences of the therapy can be observed and provide plausible mechanisms for unforeseen outcomes (e.g.treatment failure and spatial distribution of immune cells). This makes the framework suitable for simulating complex biological systems with sub-scale components (molecular, cellular, tissue, organism) and inherent emerging behaviour with limited empirical data and most relevant to elucidate unexpected behaviors (micro-known to produce macro-unknown).

Adjustments were made to simplify the model and aid in easier interpretation of the results: cells are arranged on a lattice, immune cells are simplified and the vasculature was omitted. This could come with some limitations: Having a lattice constrains the size of cells. One lattice can only accommodate a certain number of cells while in reality cancer cells could make clusters with different sizes. Cancer cells can vary in size and shape, particularly when they proliferate and form clusters or masses of differing dimensions. A lattice forces the model to enforce a uniform cell size which doesn’t capture potential heterogeneity in cell sizes [42]. The rigid lattice forces cancer cells to adhere to a regular arrangement. In reality, cancer cells can arrange themselves in arbitrary shapes and sizes, clustering together in complex, irregular patterns. Additionally, the lattice restricts neighbour interactions. Each cell can only interact with its immediate neighbors. In addition, scaling the number of cells in the simulation versus the number of cells in reality could cause inaccuracies in the cell-cell interaction. Further, in our simulation moving and placing daughter cells after division is only possible on discrete lattice points while in reality immune cells could move at a continuous distance. Off-lattice models could be used but those require additional computational power as collision and overlapping placements of cells need to be avoided. In this model immune cells are modelled as either suppressor or effector cells while in reality the TME consists of different types of cells such as CD8+ T cells, natural killer cells, regulatory T cells, and myeloid-derived suppressor cells [43]. The immune response is further simplified by omitting T cell priming, trafficking, recruiting to the TME, and cytokines. By incorporating different immune cell types and pathways, the model can better capture the full range of interactions and dynamics occurring in the TME. Different patients exhibit unique immune landscapes, with varying distributions of immune cell types in the TME. A more granular immune model would allow for the inclusion of patient-specific immune cell profiles, enabling more personalized predictions of treatment outcomes. Immunotherapies (e.g., checkpoint inhibitors, CAR-T cell therapy, and cytokine therapies) often aim to modulate the activity of specific immune cells. A more granular model can help predict the efficacy and potential side effects of immunotherapies. This could help guide decisions on which immunotherapies might be most effective, as well as identify potential biomarkers for response. Furthermore, the tumour vasculature is not simulated in the examples as immune effector cells can randomly enter the TME at a free location on the lattice. This could be addressed in future by limiting the entry points to certain locations of the grid.

Despite its simplifications, the model can capture characteristics of cancers such as growth, PDL1 expresssion rate, antigenicity, and consequences of different treatment options on the TME and cell-cell interaction and provides insight in emerging behaviour. Future work could be calibrating the model to a certain type of cancer and patient to generate predictions that could be used in clinical practice for hypothesis testing. Emerging technologies such as multiregion sequencing, single-cell sequencing, analysis of autopsy samples, and longitudinal analysis of liquid biopsy samples are potential methods to gain insight into the complex architecture of cancers [44]. The data required for model calibration would include tumour biopsies from cancer patients before and after treatment. Those data sets would include the numbers of different cell populations and their spatial distribution [17]. Due to the explicit spatial set up in our ABM, pathological images from tumour biopsies and model outputs can be compared. Those patient data could serve as initial conditions for the simulation, as well as for calibration and validation purposes. Detected objects such as cancer cells and immune cells can be mapped with the corresponding agents in model [45]. Further work could be varying parameter values to fit the model to a certain tumour type, and simulating the TME architecture in more detail such as including vasculature. Adding vasculature to a tumor model is useful to enable better simulation of spatial processes such as oxygen and nutrient supply, drug delivery, and immune cell infiltration. Lastly, based on the model framework in this setting, the process of finding the best manner of combining or scheduling treatment for a given tumour could be automatized using artificial intelligence methods such as reinforcement learning, where the dosing and scheduling of this combination can be optimised. One example is the work by Jalalimanesh *et al.* where reinforcement learning in combination with agent-based modelling is used to optimise radiotherapy [46].

## Supplementary Information

Below is the link to the electronic supplementary material.

Supplementary file 1 (pdf 310 KB)

Supplementary file 2 (pdf 2895 KB)

## Data Availability

No datasets were generated or analysed during the current study.

## References

[1] Han Y, Liu D, Li L (2020) PD-1/PD-L1 pathway: current researches in cancer. Am J Cancer Res 10(3):727 32266087 PMC7136921

[2] Nabrinsky E, Macklis J, Bitran J (2022) A Review of the Abscopal Effect in the Era of Immunotherapy. Cureus 14(9) 10.7759/cureus.29620 PMC9604762 36321062

[3] Carvalho HD, Villar RC (2018) Radiotherapy and immune response: the systemic effects of a local treatment. Clinics 73 10.6061/clinics/2018/e557s PMC6257057 30540123

[4] Alfonso JC et al (2021) Tumor-immune ecosystem dynamics define an individual Radiation Immune Score to predict pan-cancer radiocurability. Neoplasia 23(11):1110–1122 34619428 10.1016/j.neo.2021.09.003 PMC8502777

[5] Hiro Sato et al (2019) Radiotherapy and PD-L1 expression. Gan to Kagaku ryoho. Cancer & Chemotherapy 46(5):845–849 31189801

[6] Powathil GG, Adamson DJ, Chaplain MA (2013) Towards predicting the response of a solid tumour to chemotherapy and radiotherapy treatments: clinical insights from a computational model. PLoS Computat Biol 9(7):e1003120 10.1371/journal.pcbi.1003120 PMC3708873 23874170

[7] Checkley S et al (2015) Bridging the gap between in vitro and in vivo: dose and schedule predictions for the ATR inhibitor AZD6738. Sci Reports 5(1):1–12 10.1038/srep13545 PMC4550834 26310312

[8] Nehme A et al (2001) Modulation of docetaxel-induced apoptosis and cell cycle arrest by all-trans retinoic acid in prostate cancer cells. British J Cancer 84(11):1571–1576 10.1054/bjoc.2001.1818 PMC2363662 11384110

[9] Fabian KP, Wolfson B, Hodge JW (2021) From Immunogenic Cell Death to Immunogenic Modulation: Select Chemotherapy Regimens Induce a Spectrum of ImmuneEnhancing Activities in the Tumor Microenvironment. Front Oncol 11 10.3389/fonc.2021.728018 PMC8419351 34497771

[10] Ng HY et al (2018) Chemotherapeutic treatments increase PD-L1 expression in esophageal squamous cell carcinoma through EGFR/ERK activation. Translational Oncol 11(6):1323–1333 10.1016/j.tranon.2018.08.005 PMC6122398 30172884

[11] Lindauer A et al (2017) Translational pharmacokinetic/pharmacodynamic modeling of tumor growth inhibition supports dose-range selection of the anti–PD-1 antibody pembrolizumab. CPT: Pharmacometrics Syst Pharmacol 6(1):11–20 10.1002/psp4.12130 PMC5270293 27863176

[12] Truong VT et al (2021) Step-by-step comparison of ordinary differential equation and agentbased approaches to pharmacokinetic-pharmacodynamic models. Pharmacometrics & Syst Pharmacol, CPT 10.1002/psp4.12703 PMC8846629 34399036

[13] Monte UD (2009) Does the cell number 109 still really fit one gram of tumor tissue? Cell Cycle 8(3):505–506 19176997 10.4161/cc.8.3.7608

[14] Erdi YE (2012) Limits of tumor detectability in nuclear medicine and PET. Molecular Imaging Radionuclide Therapy 21(1):23 23486256 10.4274/Mirt.138 PMC3590963

[15] Norton L (1988) A Gompertzian model of human breast cancer growth. Cancer Res 48(24 Part 1):7067–7071 3191483

[16] Brunton GF, Wheldon TE (1980) The Gompertz equation and the construction of tumour growth curves. Cell Proliferation 13(4):455–460 10.1111/j.1365-2184.1980.tb00486.x 7428019

[17] Gong C et al (2017) A computational multiscale agent-based model for simulating spatiotemporal tumour immune response to PD1 and PDL1 inhibition. J Royal Soc Interface 14(134):20170320 10.1098/rsif.2017.0320 PMC5636269 28931635

[18] Onion D et al (2018) Multicomponent analysis of the tumour microenvironment reveals low CD8 T cell number, low stromal caveolin-1 and high tenascin-C and their combination as significant prognostic markers in non-small cell lung cancer. Oncotarget 9(2):1760 29416729 10.18632/oncotarget.18880 PMC5788597

[19] Xing X et al (2018) Analysis of PD1, PDL1, PDL2 expression and T cells infiltration in 1014 gastric cancer patients. Oncoimmunology 7(3):e1356144 29399387 10.1080/2162402X.2017.1356144 PMC5790386

[20] Chiba T et al (2004) Intraepithelial CD8+ T-cell-count becomes a prognostic factor after a longer follow-up period in human colorectal carcinoma: possible association with suppression of micrometastasis. British J Cancer 91(9):1711–1717 10.1038/sj.bjc.6602201 PMC2410024 15494715

[21] Hiraoka K et al (2006) Concurrent infiltration by CD8+ T cells and CD4+ T cells is a favourable prognostic factor in non-small-cell lung carcinoma. British J Cancer 94(2):275–280 10.1038/sj.bjc.6602934 PMC2361103 16421594

[22] Marques P et al (2019) Chemokines modulate the tumour microenvironment in pituitary neuroendocrine tumours. ACta Neuropathologica Commun 7:1–21 10.1186/s40478-019-0830-3 PMC6839241 31703742

[23] Cooper GM, Adams K (2023) The cell: a molecular approach. Oxford University Press

[24] Cooper GM (2000) The cell : a molecular approach. eng. 2nd ed. Washington, D.C: ASM Press. isbn: 0-87893-106-6

[25] Chow AY (2010) Cell cycle control by oncogenes and tumor suppressors: driving the transformation of normal cells into cancerous cells. Nat Educ 3(9):7

[26] Ribas A, Hu-Lieskovan S (2016) What does PD-L1 positive or negative mean? J Experimental Med 213(13):2835–2840 10.1084/jem.20161462 PMC5154949 27903604

[27] Gillespie DT (1977) Exact stochastic simulation of coupled chemical reactions. J Phys Chem 81(25):2340–2361

[28] Salem A et al (2019) Oxygen-enhaünced MRI is feasible, repeatable, and detects radiotherapyinduced change in hypoxia in Xenograft models and in patients with non-small cell lung Cancer. Clinical Cancer Res 25(13):3818–3829 31053599 10.1158/1078-0432.CCR-18-3932

[29] Rckert M et al (2021) Radiotherapy and the immune system: More than just immune suppression. Stem Cells 39(9):1155–1165 33961721 10.1002/stem.3391

[30] Frances N et al (2011) Tumor growth modeling from clinical trials reveals synergistic anticancer effect of the capecitabine and docetaxel combination in metastatic breast cancer. Cancer Chemotherapy Pharmacol 68(6):1413–1419 10.1007/s00280-011-1628-6 21476101

[31] Terranova N et al (2021) Population pharmacokinetics of ATR inhibitor berzosertib in phase I studies for different cancer types. Cancer Chemotherapy Pharmacol 87:185–196 10.1007/s00280-020-04184-z PMC7870753 33145616

[32] Topalian SL et al (2016) Mechanism-driven biomarkers to guide immune checkpoint blockade in cancer therapy. Nat Rev Cancer 16(5):275–287 27079802 10.1038/nrc.2016.36 PMC5381938

[33] Ilie M et al (2016) Comparative study of the PD-L1 status between surgically resected specimens and matched biopsies of NSCLC patients reveal major discordances: a potential issue for anti-PD-L1 therapeutic strategies. Ann Oncol 27(1):147–153 26483045 10.1093/annonc/mdv489

[34] Lyford-Pike S et al (2013) Evidence for a role of the PD-1: PD-L1 pathway in immune resistance of HPV-associated head and neck squamous cell carcinoma. Cancer Res 73(6):1733–1741 23288508 10.1158/0008-5472.CAN-12-2384 PMC3602406

[35] Mietz J et al (2024) Human effector CD8+ T cells with an activated and exhausted-like phenotype control tumour growth in vivo in a humanized tumour model. Ebiomedicine 106 10.1016/j.ebiom.2024.105240 PMC11296066 38986249

[36] Thomas F et al (1988) Radiotherapy alone for oropharyngeal carcinomas: the role of fraction size (2 Gy vs 2.5 Gy) on local control and early and late complications. Int J Radiation Oncol* Biol* Phys 15(5):1097–1102 10.1016/0360-3016(88)90190-3 3182341

[37] Kupelian PA et al (2005) Hypofractionated intensity-modulated radiotherapy (70 gy at 2.5 Gy per fraction) for localized prostate cancer: long-term outcomes. Int J Radiation Oncol* Biol* Phys 63(5):1463–1468 10.1016/j.ijrobp.2005.05.054 16169683

[38] Stuschke M, Pöttgen C (2004) Localized small-cell lung cancer: which type of thoracic radiotherapy and which time schedule. Lung Cancer 45:S133–S137 15552793 10.1016/j.lungcan.2004.07.981

[39] Carlos-Reyes A et al (2021) Biological adaptations of tumor cells to radiation therapy. Front Oncol 11:718636 34900673 10.3389/fonc.2021.718636 PMC8652287

[40] Demaria S, Formenti SC (2012) Role of T lymphocytes in tumor response to radiotherapy. Front Oncol 2:95 22937524 10.3389/fonc.2012.00095 PMC3426850

[41] Galluzzi L et al (2023) Emerging evidence for adapting radiotherapy to immunotherapy. Nat Rev Clinical Oncol pp 1–15 10.1038/s41571-023-00782-x 37280366

[42] Wu PH et al (2015) Evolution of cellular morpho-phenotypes in cancer metastasis. Sci Reports 5(1):18437 10.1038/srep18437 PMC4682070 26675084

[43] Xing Y et al (2021) Tumor immune microenvironment and its related miRNAs in tumor progression. Front Immunol 12:624725 34084160 10.3389/fimmu.2021.624725 PMC8167795

[44] Dagogo-Jack I, Shaw AT (2018) Tumour heterogeneity and resistance to cancer therapies. Nat Rev Clinical Oncol 15(2):81–94 29115304 10.1038/nrclinonc.2017.166

[45] Gong C et al (2019) Quantitative characterization of CD8+ T cell clustering and spatial heterogeneity in solid tumors. Front Oncol 8:649 30666298 10.3389/fonc.2018.00649 PMC6330341

[46] Jalalimanesh A et al (2017) Simulation-based optimization of radiotherapy: Agent-based modeling and reinforcement learning. Math Comput Simulation 133:235–248

