# Supplementary material for: A hybrid PKPD agent-based model of the tumour immune interaction: effects of anti-cancer combination therapy
Source: J Pharmacokinet Pharmacodyn. 2026 Mar 24;53(3):16. doi: 10.1007/s10928-026-10021-2 (PMC13013190; doi:10.1007/s10928-026-10021-2)
Supplement: Supplementary file 1 — (pdf 310 KB) [file 10928_2026_10021_MOESM1_ESM.pdf]

A hybrid PKPD agent-based model of the tumour immune interaction: effects of anti-cancer combination therapy

Van Thuy Truong[1,2], Grant Lythe[2], Paolo Vicini[3],
James W. T. Yates[4], Vincent F. S. Dubois[1]

Corresponding author: Van Thuy Truong, School of Mathematics, University of Leeds, Woodhouse, Leeds LS2 9JT, vn.thuy.truong@gmail.com

Affiliations:

[1] Clinical Pharmacology and Quantitative Pharmacology, Clinical Pharmacology and Safety Sciences, AstraZeneca, Aaron Klug Building, Granta Park, Cambridge, CB21 6GH, UK

[2] Department of Applied Mathematics, University of Leeds, Leeds, United Kingdom

[3] Confo Therapeutics, Technologiepark 94, 9052 Ghent (Zwijnaarde), Belgium

[4] DMPK, IVIVT, RD Research, GSK , Gunnels Wood Road, Stevenage, Hertfordshire, SG1 2NY, United Kingdom

1 Supplementary part 1: model implementation

1.1 Oxygen in the environment

Oxygen in the TME diffuses with coefficient D and is consumed by cancer cells. The oxygen concentration $U(x, y, z)$ depends on the spatial variables x, y, z. The PDE can be written as

$$\frac{\partial U}{\partial t} = D\left(\frac{\partial^2 U}{\partial x^2} + \frac{\partial^2 U}{\partial y^2} + \frac{\partial^2 U}{\partial z^2}\right) - k_o U \epsilon_o - k_o q_c U \epsilon_c. \tag{1}$$

The factor ϵ_0 is equal to 1 if there is a non-quiescent cancer cell within one grid distance of (x, y, z) and is equal to 0 otherwise; the factor ϵ_c is equal to 1 if there is a quiescent cell within one grid distance of $(x, y, z$ and is equal to 0 otherwise. We generate solutions using forward Euler in time and second-order central finite differences.

Parameter	Value
Diffusion coefficient D	1 [μm^2/d]
Division range	$100 - 50\%$
Quiescence range	$50 - 30\%$
Death range	$30 - 0\%$
Oxygen consumption rate for dividing cancer cells k_o	0.001 [%/d]
Factor for oxygen consumption of quiescent cells q_c	0.01

Table 1: Parameters for oxygen diffusion and consumption.

1.2 PD1 antibody

The PD1 antibody treatment module is implemented with a PKPD model from the literature [1] where a system of ODEs is used to calculate the receptor occupancy of pembrolizumab on the PD1 effector cell receptor which will determine the effector cell exhaustion rate.

Equation 2 describes the central compartment. The monoclonal PD1 antibody is administrated i.v. into the central (blood) compartment as the concentration C_1, where it can be distributed to the peripheral compartment as the concentration C_2 and redistributed to the central compartment with rates K_{12} (flux rate from C_1 to C_2), K_{21} (flux rate from C_2 to C_1). Elimination from the central compartment is with the rate K or with the Michaelis-Menten rate $\frac{V_{max}}{K_M+C_1}$. Inside the central compartment, the antibody can bind to the PD1 receptors in the blood (e.g on immune effector cells) denoted with C_{PD1b} which leads to the bound drug-receptor complex $PD1_b$. Binding and detaching rates are K_{onPD1} and K_{offPD1}. The drug in the central compartment is distributed to and redistributed back from the vascular space of the tumour as C_{vs} with the rate PLQ. The peripheral compartment is represented by equation 3 where the drug from the central compartment C_1 is distributed to the peripher compartment with the efflux rate K_{12} and the drug in the peripheral compartment C_2 is redistributed to the central compartment with the rate K_{21}.

The tumour plasma flow transports the unbound antibody to the tumour compartment at the rate PLQ. The tumour compartment consists of the vasculature, the endosomal layer and the interstitium. Equation 4 describes the drug C_{vs} in the vascular space of the tumour which is transported into the vasculature with the rate PLQ from the central compartment. The lymph flow can eliminate the drug from the vascular compartment with the rate L or transport it into the interstitium with the rate $(1 - vref) * L$.

The monoclonal antibody inside the vasculature (C_{vs}) is transported from the tumour vasculature to the interstitium via the neonatal fragment crystallizable-receptor (FcRn) salvage pathway [2]. The antibody will be engulfed and released by the endosomal cell through pinocytosis with the rate $CLup$. Pinocytosis can also transport drug back into the vasculature from the endosomal space with the rate $FR * CLup$. Equation 5 describes the fate of the antibody in the endosomal space. Pinocytosis transport the drug from the vasculature (C_{vs}) and the intestitium (C_{is}) into the endosomal space. Inside the endosomal space the unbound drug (C_{ub}) binds and bound drug (C_b)

unbinds to the FcRn receptor with the rates K_{onFcRn} and $K_{offFcRn}$. Unlike unbound proteins or unbound antibodies (C_{ub}), the FcRn receptor antibody complex will not be degraded by lysosomes with the rate K_{deg}. The bound drug's fate is described with equation 6. A fraction of the bound drug (C_b) on the FcRn receptor will be released by the vesicle to either the vascular side as C_{vs} or the interstitial side as C_{is} with the rate $FR * CLup$. Attachment and detachment of unbound and bound drug (C_b and C_{ub}) to the receptor $FcRn$ happens with the rates K_{onFcRn} and $K_{offFcRn}$.

Equation 7 describes the kinetics of the FcRn receptor. Drug from the vasculature C_{vs} is transported to the FcRn receptor with the rate CL_{up}. Attachment and detachment of unbound and bound drug (C_b and C_{ub}) to the receptor $FcRn$ happens with the rates K_{onFcRn} and $K_{offFcRn}$. Inside the interstitial compartment (equation 8) drug is transported into that compartment with the lymph flow with the rate $(1 - vref) * L$ from the vasculature as C_{vs} or the intestitium as C_{is}. Additionally, pinocytosis contributes to drug leaving the intestitum with the rate CL_{up} and bound drug C_b entering from the endosomal space with the rate $CL_{up} * (1 - FR)$. The antibody concentration C_{is} binds and unbinds to the PD1 receptor on immune effector cells ($CPD1_t$) with the rates K_{onPD1} and K_{offPD1}. The bound drug-receptor complex inside the tumour $PD1_t$ can be degraded with the rate K_{degPD1} (see equation 9). Similarly, the bound drug-receptor complex inside the blood compartment (see equation 10), attaches and detaches with the rates K_{onPD1} and K_{offPD1} and degrades with rate K_{degPD1}. The bound drug-receptor complex $PD1_t$ initiates hyperbolic feedback and cause a receptor upregulation ($MPD1_t$) on the immune effector cell (see equation 11). Additionally, the receptor on the immune cell $MPD1_t$ is eliminated with the rate k_{out}. In equation 12 the receptor occupancy of the drug in the tumour compartment is calculated with the relation of antibody-drug complex ($PD1_t$) to the concentration of unoccupied receptors ($CPD1_t$).

Central compartment:

$$\begin{aligned} V_1 * \frac{dC_1}{dt} &= -K * (C_1 * V_1) - \frac{V_{max}}{K_M + C_1} * C_1 - PLQ * C_1 + PLQ * C_{vs} - K_{12} * C_1 * V_1 \\ &+ K_{21} * C_2 * V_2 - K_{onPD1} * (C_{PD1_b} - PD1_b) + K_{offPD1} * PD1_b * V_1 \end{aligned} \tag{2}$$

Peripheral compartment:

$$V_2 * \frac{dC_2}{dt} = K_{12} * C_1 * V_1 - K_{21} * C_2 * V_2 \tag{3}$$

Vascular space tumour:

$$\begin{aligned} V_{vs} * \frac{dC_{vs}}{dt} &= PLQ * C_1 - (PLQ - L) * C_{vs} - (1 - v_{ref}) * L * C_{vs} \\ &- CL_{up} * C_{vs} + CL_{up} * FR * C_b \end{aligned} \tag{4}$$

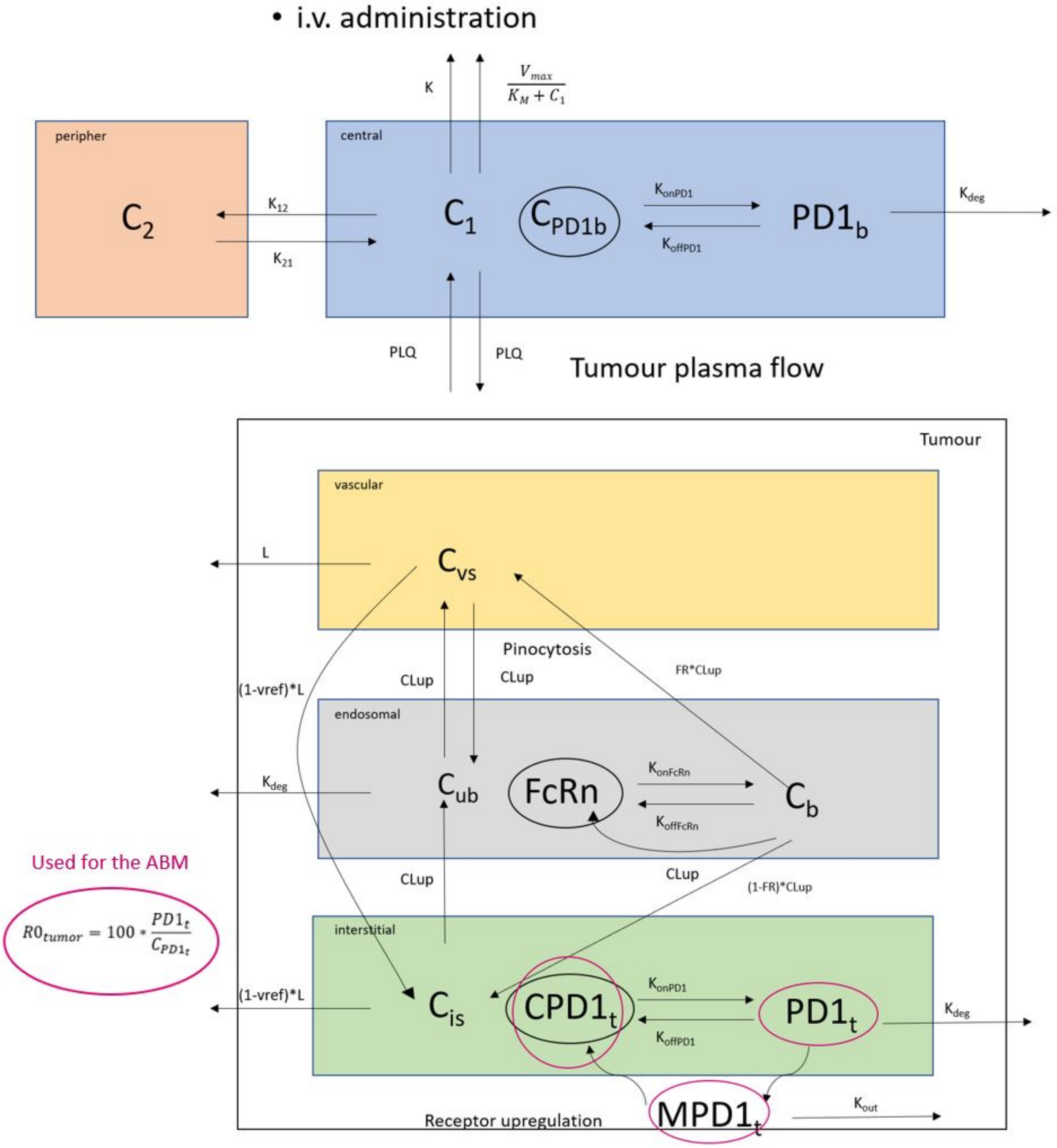

Figure 1: Schematic of the PKPD model for pembrolizumab

Endosomal space monoclonal antibody (mAb) unbound to FcRn:

$$\frac{dC_{ub}}{dt} = \frac{CL_{up}}{V_{es}} * (C_{vs} + C_{is}) - K_{onFcRn} * C_{ub} * FcRn + K_{offFcRn} * C_b - K_{deg} * C_{ub} \tag{5}$$

Endosomal space mAb bound to FcRn:

$$\frac{dC_b}{dt} = -\frac{CL_{up}}{V_{es}} * C_b + K_{onFcRn} * C_{ub} * FcRn - K_{offFcRn} * C_b \tag{6}$$

Endosomal FcRn:

$$\frac{dFcRn}{dt} = \frac{CL_{up}}{V_{es}} * C_{vs} - K_{onFcRn} * C_{ub} * FcRn + K_{offFcRn} * C_b \tag{7}$$

Interstitial compartment:

$$\begin{aligned} V_{is} * \frac{dC_{is}}{dt} &= (1 - vref) * L * C_{vs} - (1 - vref) * L * C_{is} - CL_{up} * C_{is} \\ &+ CL_{up} * (1 - FR) * C_b - K_{onPD1} * C_{is} * V_{is} * (C_{PD1_t} - PD1_t) + K_{offPD1} * PD1_t * V_{is} \end{aligned} \tag{8}$$

Drug receptor binding in the tumour:

$$\frac{dPD1_t}{dt} = K_{onPD1} * C_{is} * (C_{PD1_t} - PD1_t) - K_{offPD1} * PD1_t - K_{degPD1} * PD1_t \tag{9}$$

Drug receptor binding in blood:

$$\frac{dPD1_b}{dt} = K_{onPD1} * C_1 * (C_{PD1_b} - PD1_b) - K_{offPD1} * PD1_b - K_{degPD1} * PD1_b \tag{10}$$

Tumour PD-1 receptor upregulation and elimination (amount):

$$\frac{dMPD1_t}{dt} = k_{in} * \left(1 + E_{maxtp} * \frac{PD1_t}{EC_{50tp} + PD1_t}\right) - k_{out} * M_{PD1t} \tag{11}$$

Receptor occupancy:

$$R0_{tumour} = 100 * \frac{PD1_t}{C_{PD1_t}} \tag{12}$$

1.3 Radiotherapy

In the radiotherapy module, the survival probability of each cell is simulated with a modified linear quadratic model and oxygen modification factor (OMF) according to Powathil et al [3].

$$OMF = \frac{OER * (pO_2)}{OER_m} = \frac{1}{OER_m} * \frac{OER_m * pO_2(x) + K_m}{pO_2(x) + K_m} \tag{13}$$

$pO_2(x)$ describes the oxygen concentration at position x. The ratio of the radiation doses needed for the same cell kill under anoxic and oxic conditions is OER. $OER_m = 3$ is the maximum ratio. K_m is defined as the pO2 which induces half of the maximum OER, this value is fixed to 3mm Hg.

The modified linear quadratic model is used to calculate the survival probability of each cell:

$$S(d) = exp[\gamma * (-\alpha * OMF * d - \beta * (OMF * d)^2)] \tag{14}$$

S(d) describes the survival probability of a cell which has a certain oxygen modification factor due to its position and receives a radiation dose d. α and β are sensitivity parameters. The sensitivity parameters γ reflect the different susceptibility of each cell kind and cancer cell cycle phase.

$$\gamma = \begin{cases} 1 & \text{for cancer cells in S-G2-M phase [3]} \\ 0.5 & \text{for cancer cells in the G1 phase [3]} \\ 0.25 & \text{for cancer cells in the G0 phase [3]} \\ 1 & \text{for immune effector cells [assumed]} \\ 0.6 & \text{for immune suppressor cells [assumed]} \end{cases} \tag{15}$$

The sensitivity parameter γ ranges from 0 to 1. The S, G2, and M phases are the most radiosensitive phases with $\gamma = 1$, while the resting phase G0 is the least affected phase by radiation with $\gamma = 0.25$. The G1 phase has a γ value of 0.5. Immune effector cells are more radiosensitive than immune suppressor cells [4]. Hence, it is assumed that γ is 1 for effector cells and 0.6 for suppressor cells.

Considering that damage caused by low-dose radiation (less than 5 Gy), can be repaired within hours, a modified linear quadratic model is used [3].

$$S^*(d) = \begin{cases} S & d > 5 \text{ Gy} \\ S + (1 - S) * 0.5 & d \leq 5 \text{ Gy} \end{cases} \tag{16}$$

To reflect the faster repair for doses smaller than 5 Gy, the survival probability is higher by $(1 - S) * 0.5$ in comparison to doses bigger than 5 Gy. The cell cycle delay in the G1 and G2

phase after radiation is drawn from a uniform distribution between 1-9h [3]. A cell survives if a random number drawn from a uniform distribution between 0-1 is smaller than the modified survival probability $S^*(d)$ and dies otherwise. Immune cell infiltration is modelled deterministically according to Alfonso et al [5] and depends on the number of radiotherapy doses N_{RT}, time of irradiation t_j, number of cancer cells killed by the irradiation K_{Tj}, a stimulation decay c, and immune cell recruitment factors δ_E for immune effector cells and δ_S for suppressor cells.

Immune cell infiltration:

$$E_i =_{j=1}^{N_{RT}} K_{Tj} * (\delta_E * e^{-c*(t_i - t_j)}) \tag{17}$$

$$S_i =_{j=1}^{N_{RT}} K_{Tj} * (\delta_S * e^{-c*(t_i - t_j)}) \tag{18}$$

1.4 Chemotherapy

For the chemotherapy module, we simulated treatment with docetaxel with a K/PD model from Frances et al [6].

$$\frac{dD}{dt} = -k_d * D + u_D(t) * D(0) \tag{19}$$

where D describes the docetaxel amount, k_D the 'biological constant' to control the dose history profiles, $u_D(t)$ the dosing schedule, and D(0) the initial dose. To take into consideration that the drug distribution differs throughout the 3D lattice, the drug concentration is scaled based on the predicted oxygen gradient. To take emerging resistance into account, the kill rate $f_D(t)$ depends on the time t, the amount of drug in the system $D(t)$, the efficacy rate p_D, and the resistance parameter r_D.

$$f_D(t) = p_D * e^{(-r_D * t)} * D(t) \tag{20}$$

The chemotherapy will affect immune cells and cancer cells in the G2 and M phases. The death probability is calculated by the kill rate $f_D(t)$ divided by the maximum kill rate. A random number is drawn from a uniform distribution, if it is smaller than the survival probability, the cell with die and survive otherwise.

1.5 DNA damage response inhibitor

The DNA damage response inhibitor treatment module is based on a PKPD model from Terranova et al [7].

$$\frac{dCEN}{dt} = -(q + cl) * \frac{CEN}{v1} + q * \frac{PER}{v2} \tag{21}$$

$$\frac{dPER}{dt} = q * (\frac{CEN}{v1} - \frac{PER}{v2}) \tag{22}$$

where CEN is the drug amount in mg/m^2 given i.v. in the central compartment. From there the drug amount can be distributed and redistributed to the peripheral compartment with the rate q where it will be the drug amount PER (see equation 21). The drug is cleared from the body with the rate cl (see equation 21). $v1$ is the volume of the central compartment and $v2$ is the volume of the peripheral compartment. The drug in the peripheral compartment PER is described in equation 22. Distribution and redistribution of drug from and to the central compartment happen with rate q. To take into account that the drug that reaches the cancer cells depends on the location, the drug amount predicted by the PK ODE model is scaled based on the predicted oxygen gradient. The drug effect $E(\bar{x}, t)$ at time t and at a certain location $\bar{x}$ is modelled by using the Emax model

$$E(\bar{x}, t) = Emax * \frac{CEN(\bar{x}, t)^h}{EC_{50}^h + CEN(\bar{x}, t)^h} \tag{23}$$

where $Emax$ denotes the maximal drug effect. Emax is 1 when the repair inhibition is complete. EC_{50} is the drug concentration that archives half of the maximal drug effect ($E_{max}/2$) and h is the Hill coefficient.

The drug only affects cancer cells in the S phase. A random number is drawn and compared with survival probability $E(\bar{x}, t)$. If that number is bigger than $E(\bar{x}, t)$, the cell dies.

1.6 Code implementation in python

The model is programmed in the python language. The python code can be found at

https://github.com/VanThuyTruong/ABM_PKPD.

1.6.1 Stochastic agent based model

The individual agents are instances of a python class. Each class of agents (cancer cells, immune effector cells, immune suppressor cells) have their own attributes (for example: age, position, time to division, or PDL1 status). Those attributes are coded as class attributes. Each class has actions defined in the class object (for example: move, update cell cycle or consume oxygen). The code is run for a certain time (in this paper for around 300 days). The actions are chosen according to the Gillespie algorithm [8, 9] where a random number is drawn to chose an action. The probabilities of the action are set by rates multiplied by certain cell numbers. Cancer cell division happens with the division rate times the number of existing cancer cells in the environment which makes division more likely the higher the cancer cells numbers are. A PDL1 expression event is more likely when more cancer cells are being eliminated by effector cells, chemotherapy or radiotherapy. The cancer kill probability increases with the number of effector cells while the natural cancer cell death rate depends on the number of cancer cells. The immune effector infiltration rate increases with the number of cancer cells, immune effector cells in the TME and eliminated cancer cells by the immune reaction while the suppressor infiltration rate depends on the number of tumour cells that have been killed by effector cells. Immune cell division and moving rates are higher with a larger number of immune cells. The effector exhaustion rate depends on the number of suppressor cells, PDL1+ cancer cells and the receptor occupancy with the antibody. The time step is set

according to the Gillespie algorithm by creating the negative log of a random number between 0 to 1. This number is divided by the sum of the rates which ensures that the time steps are smaller if more agents are in the simulation.

1.6.2 Combining ODEs with the agent based model

The ODEs are solved with the solver scipy.integrate.odeint from the python scipy package. The initial dose is set as the initial condition and a start time is chosen. The ODE is solved per timestep of the agent based model with the dose at the end of the last time step as the new initial condition. In case of multiple dose the new initial dose is the new dose plus the remaining concentration from the previous time step as the new initial condition. Agents such as the tumour cells are affected by the drug concentration at a specific time point in the simulation.

1.6.3 Combining the PDE with the agent based model

The agent based model is simulated on a 100x100x100 grid which is used to compute the PDE. The boundary condition is 1 as on the boundaries there are no cancer cells to consume oxygen and to simulate that oxygen diffuses from the periphery to the tumour. The values for the oxygen are set as 1 to 0 with 1 as 100% oxygen availability which decreases when cancer cells grow and consume oxygen. Hence, the initial condition is 1. As the tumour grows, new cancer cells consume oxygen on the grid point where they are located, and the oxygen grid updated with new values. The PDE is solved per time step of agent based model. If time steps are too small, then the PDE is not solved every time step but for example for every 10th time step to save computational power. Depending on the oxygen value on their grid point, cancer cells remain active or go into the quiescence state or necrosis. For the chemotherapy and DNA damage response inhibitor, the drug concentration is multiplied with the oxygen number (ranged from 1 to 0) on the PDE grid. This simulates that in the tumour core less drug is available similarly to having less oxygen diffusion in the middle of the tumour. The PD1 antibody concentration is not multiplied with the PDE values as the immune effector cells come from the periphery to the tumour. Hence, the antibody concentration they are exposed to is high.

Parameter	**Value**	**Units**	**Source**
**Reaction rates (Gillespie algorithm)**			
Cancer cell division rate	0.01		assumed
Cancer cell mutation rate	0.009		assumed
Cancer cell kill rate	0.01		assumed
Cancer cell death rate	0.0001		assumed
Suppressor cell moving rate	0.02		assumed
Suppressor cell division rate	0.02		assumed
Suppressor cell infiltration rate	0.0005		assumed
Effector cell exhaustion rate	0.01		assumed
Effector cell moving rate	0.02		assumed
Effector cell division rate	0.01		assumed
Effector cell infiltration rate	0.005		assumed
**Cell attributes**			
**Cancer cells**			
Initial number	100		assumed
Time to division	24	h	assumed
**Suppressor cells**			
Initial number	1		assumed
Time to division	8	h	assumed
Division count	8		assumed
Lifespan	3*24	h	assumed
**Effector cells**			
Initial number	1		assumed
Time to division	8	h	[10]
Division count	8		[10]
Lifespan	3*24	h	[10]
**Oxygen PDE**			
Diffusion coefficient D	1	$\mu m^2/d$	assumed
Division range	100-50	%	assumed
Quiescence range	50-30	%	assumed
Death range	30-0	%	assumed
Oxygen consumption rate for dividing cancer cells k_o	0.001	$\%/d$	assumed
Factor for oxygen consumption of quiescent cells q_c	0.01		assumed
**PD1 antibody treatment**			
Molecular weight	149000	g/mol	[11]
V1	2877/1000	l	[11]
V2	2854/1000	l	[11]
W0=V_tot	170/1000000	l	[11]
V_max	114/(MW)*1e3	nmol/h	[11]
V_es	0.005*V_tot	l	[11]
V_is	0.55*V_tot	l	[11]
K_M	0.078/(MW)*1e6	nmol/l	[11]
PLQ	12.7*V_vs	l/h/l	[11]
L	0.002*PLQ*V_vs	l/h/l	[11]
Q	384/(1000*24)	l/h	[11]

K_12	Q/V_1	1/h	[11]
K_21	Q/V_2	1/h	[11]
K_off_PD1	0.144	1/h	[11]
Kon_PD1_iv	2880e6/1e9	1/M/h	[11]
K_IVIV	1		[11]
K_on_PD1= Kon_PD1_iv/K_IVIV		1/nM/h	[11]
CL	167/(1000*24)	l/h	[11]
K	CL/V_1	1/h	[11]
N_Tcell	1000	number of T cells per μl blood	[11]
T_multi	4.3	initial ratio target concentration in tumour vs blood	[11]
V_blood	1400	μl	[11]
N_PD1_TC	10000	number of PD1 receptor per T cell	[11]
N_PD1_b	N_PD1_TC * N_Tcell * V_blood	number of PD1 in blood	[11]
M_PD1_b	N_PD1_b/AV*1e9	PD1 amount in nmoles in blood	[11]
C_PD1b	M_PD1_b/V_1	nmol/l	[11]
C_PD1t	T_multi*C_PD1b	nmol/l	[11]
vref	0.842		[11]
vref_is	0.2		[11]
CL_up	0.0366*V_es	l/h/l	[11]
FR	0.715		[11]
K_on_FcRn	792e6/(1e9)	1/nM/h	[11]
K_off_FcRn	23.9	1/h	[11]
K_deg	42.9	1/h	[11]
K_deg_PD1	0.00249	1/h	[11]
BW (human)	80	kg	[11]
Dose	2	mg/kg	[11]
Initial conditions			
C_1_0	D[nmol]/V_1	nmol/l	[11]
C_2_0	0	nmol/l	[11]
C_vs_0	0	nmol/l	[11]
C_ub_0	0	nmol/l	[11]
C_b_0	0	nmol/l	[11]
FcRn_0	49.8*1000	nmol/l	[11]
C_is_0	0	nmol/l	[11]
PD1_t_0	0	nmol/l	[11]

PD1_b_0	0	nmol/l	[11]
R0t_0	0	%	[11]
M_PD1_t_0	C_PD1b*T_multi*V_is_in	nmol	[11]
EMAXTP	94.7	%	[11]
EC50TP	1.46	nM	[11]
kout	K_deg_PD1	1/h	[11]
kin	M_PD1_t_0 * kout	nmol/h	[11]
**Radiotherapy**			
OER_m	3		[3]
Km	3		[3]
d	2.5	Gy	[3]
α	0.3		[3]
β	0.3		[3]
**Immune cell infiltration**			
δ_E	0.05	1/h	[5]
δ_S	0.01	1/h	[5]
c	0.05	1/h	[5]
**Chemotherapy**			
k_d	0.0285	1/day	[6]
$D(0)$	0.132	g	[6]
p_D	0.047714		[6]
r_D	0.01251		[6]
**DNA damage response inhibitor**			
q	295	l/h	[7]
cl	65	l/h	[7]
v1	118	l	[7]
v2	1030	l	[7]
Emax	1		assumed
h	1		assumed
EC50	100.84	mg	[7]

Table 2: Model parameters of the tumour immune interaction hybrid PKPD agent based model

References

[1] J Elassaiss-Schaap et al. "Using model-based "learn and confirm" to reveal the pharmacokinetics-pharmacodynamics relationship of pembrolizumab in the KEYNOTE-001 Trial". In: *CPT: pharmacometrics & systems pharmacology* 6.1 (2017), pp. 21–28.

[2] Josiah T Ryman and Bernd Meibohm. "Pharmacokinetics of monoclonal antibodies". In: *CPT: pharmacometrics & systems pharmacology* 6.9 (2017), pp. 576–588.

[3] Gibin G Powathil, Douglas JA Adamson and Mark AJ Chaplain. "Towards predicting the response of a solid tumour to chemotherapy and radiotherapy treatments: clinical insights from a computational model". In: *PLoS computational biology* 9.7 (2013), e1003120.

[4] Shu Liu et al. "Effects of radiation on T regulatory cells in normal states and cancer: mechanisms and clinical implications". In: *American journal of cancer research* 5.11 (2015), p. 3276.

[5] Juan CL Alfonso et al. "Tumor-immune ecosystem dynamics define an individual Radiation Immune Score to predict pan-cancer radiocurability". In: *Neoplasia* 23.11 (2021), pp. 1110–1122.

[6] Nicolas Frances et al. "Tumor growth modeling from clinical trials reveals synergistic anticancer effect of the capecitabine and docetaxel combination in metastatic breast cancer". In: *Cancer chemotherapy and pharmacology* 68.6 (2011), pp. 1413–1419.

[7] Nadia Terranova et al. "Population pharmacokinetics of ATR inhibitor berzosertib in phase I studies for different cancer types". In: *Cancer Chemotherapy and Pharmacology* 87 (2021), pp. 185–196.

[8] Daniel T Gillespie. "Exact stochastic simulation of coupled chemical reactions". In: *The journal of physical chemistry* 81.25 (1977), pp. 2340–2361.

[9] Van Thuy Truong et al. "Step-by-step comparison of ordinary differential equation and agent-based approaches to pharmacokinetic-pharmacodynamic models". In: *CPT: Pharmacometrics & Systems Pharmacology* (2021).

[10] Chang Gong et al. "A computational multiscale agent-based model for simulating spatio-temporal tumour immune response to PD1 and PDL1 inhibition". In: *Journal of the Royal Society Interface* 14.134 (2017), p. 20170320.

[11] A Lindauer et al. "Translational pharmacokinetic/pharmacodynamic modeling of tumor growth inhibition supports dose-range selection of the anti–PD-1 antibody pembrolizumab". In: *CPT: pharmacometrics & systems pharmacology* 6.1 (2017), pp. 11–20.
