# Supplementary material for: A hybrid PKPD agent-based model of the tumour immune interaction: effects of anti-cancer combination therapy
Source: J Pharmacokinet Pharmacodyn. 2026 Mar 24;53(3):16. doi: 10.1007/s10928-026-10021-2 (PMC13013190; doi:10.1007/s10928-026-10021-2)
Supplement: Supplementary file 2 — (pdf 2895 KB) [file 10928_2026_10021_MOESM2_ESM.pdf]

A hybrid PKPD agent-based model of the tumour immune interaction: effects of anti-cancer combination therapy

Van Thuy Truong[1,2], Grant Lythe[2], Paolo Vicini[3],
James W. T. Yates[4], Vincent F. S. Dubois[1]

Corresponding author: Van Thuy Truong, School of Mathematics, University of Leeds, Woodhouse, Leeds LS2 9JT, vn.thuy.truong@gmail.com

Affiliations:

[1] Clinical Pharmacology and Quantitative Pharmacology, Clinical Pharmacology and Safety Sciences, AstraZeneca, Aaron Klug Building, Granta Park, Cambridge, CB21 6GH, UK

[2] Department of Applied Mathematics, University of Leeds, Leeds, United Kingdom

[3] Confo Therapeutics, Technologiepark 94, 9052 Ghent (Zwijnaarde), Belgium

[4] DMPK, IVIVT, RD Research, GSK , Gunnels Wood Road, Stevenage, Hertfordshire, SG1 2NY, United Kingdom

1 Supplementary part 2: additional simulations

1.1 Sensitivity analysis

A local sensitivity analysis is conducted to show how the tumour growth changes with different parameters. This will be useful find out which parameter is most appropriate to calibrate and to show which parameters treatment needs to influence to change the tumour immune interaction and growth dynamics.

Figures 1a-1e show how changes in the natural cancer death rate affect tumour growth and tumour immune interactions. In all cases the tumour keeps growing until approximatly 120-130 days. Then the tumour is large enought to attract immune cells. The immune cells kill the cancer cells. This cause a plateau in the cancer cell number for approximately 30 days (a plateau of around 2200 cancer cells between 130-160 days in the case of a death rate of 0.001 in Figure 1a, a plateau of approximately 3000 cancer cells from around 120-150 days for the death rate of 0.0001 in Figure 1b, a plateau of around 3000 cancer cells between day 130-150 in Figure 1c with a death rate of 0.00001, a plateau of approximately 3000 cancer cells from around 120-150 days for the death rate of 0.000005 in Figure 1d, a plateau of approximately 3000 cancer cells from around 120-150 days for the death rate of 0.0000001 in Figure 1e). Afterwards, the cancer cells start to become PDL1 positive which cause a rise of exhausted effector cells and a cancer population growth again (see

Figures 1a-1e after around 150 days) as PDL1 positive cancer cells cause effector cell exhaustion and less killing for cancer cells. With a higher cancer death rate (Figure 1a) the cancer population reaches a cell number of around 10000 cells at time point 250 days while with the lower death rates (Figures 1b-1e) the cancer cell number at time 250 days is around 25000. The differences between Figures 1b-1e are due to the stochasticity of the agent-based simulation. It can be seen that lowering the death rate beyond 0.0001 has no difference on the growth dynamics as the cancer death rate for the Gillespie algorithm gets so small that this reaction is not being chosen often.

Figures 2a-2e show how the cancer cells division rate affects the the tumour growth and tumour immune interaction. With a low cancer divion rate of 0.0001 (Figure 2a) the cancer cell number of initally 50 cells grows slowly to around 65 cancer cells at the end of simulation at day 250, very few immune effector cells (less than 5 over the course of 250 days) are in the tumour microenvironment. A higher cancer cell division rate of 0.001 (Figure 2b) causes a tumour growth from initially 50 cells to around 600 cells at 250 days. Towards the end of the simulation at at day 250 more immune effector cells are attracted to the tumour microenvironment (around 20 immune effector cells at time 250 day versus around 5 immune effector cells between 50 till 100 days). This is due to the increased cancer cell number which attracts more immune cell infiltration. A cancer cell division rate of 0.01 (Figure 2c) causes a cancer cell number of approximately 25000 at the end of simulation at 250 days. As seen in the Figures 1a-1e, the cancer growth attracts enough immune effector cells around day 120 to cause a plateau and slow down the cancer growth (around 3000 cancer cells in the plateau between day 120-150) as the immune effector cells are killing the cancer cells. At around day 150 there enough cancer cells have mutated and are PDL1 positive. That slows down the elimination of cancer cells by immune effector cells and the cancer cells population grows again. At the end of simulation at day 250, there are around 25000 cancer cells. Increasing the cancer cell division rate to 0.05 in Figure 2d causes a faster growth of the cancer cell population where 40000 cancer cells are reached at the end of simulation around day 90. The cancer cells are attracting immune effector cells earlier than in the case in Figure 2c. This will cause an earlier elimination of cancer cells by immune cell and an earlier mutation to PDL1 positive cancer cells at around 40 days with the division rate of 0.05 (Figure 2d) versus an appearance of PDL1 positive cancer cells around day 140 in the case with a division rate of 0.01 (Figure 2c). In Figure 2e the cancer division rate has increased to 0.1. It can be seen that the growth is much faster, 40000 cancer cells are reached at around 60 days (versus 40000 cancer cells being reached around day 90 with a cancer division rate of 0.05 in Figure 2d). Additionally, cancer cell mutation to PDL1 positive cells happens around 25 days with a cancer division rate of 0.1 versus PDL1 positive mutation around day 40 with a division rate of 0.05 (Figure 2d) and around day 120 with a division rate of 0.01 (Figure 2c). The number of exhausted cells increases with the division rate (around 3000 exhausted immune effector cells at day 250 with a division rate of 0.01 in Figure 2c versus around 15000 exhausted immune effector cells at day 90 with a division rate of 0.05 in Figure 2d, versus around 20200 exhausted immune effector cells at day 60 with a division rate of 0.1 in Figure 2e).

Figures 3a-3c investigate how changing the cancer killing rate affects the overall tumour immune interactions. Starting with a cancer killing rate by immune effector cells of 1 in Figure 3a, it can be seen that the cancer growth slows down around day 80 to a plateau of around 10000

cancer cells because enough immune effector cells have infiltrated and start to slow down the cancer growth by eliminating cancer cells. At the same time, cancer cells start to mutate and become PDL1 positive. The PDL1 positive cancer cells can exhaust the immune effector cells which slows down the cancer cell elimination by immune effector cells. But around 110 days all cancer cells have become PDL1 positive. The cancer population growth resumes and reaches 40000 cancer cell by day 160. Lowering the cancer killed rate to 0.1 in Figure 3b shows similar dynamics but with a delay in time. A plateau in the cancer cell growth emerges from around day 120 till day 150. PDL1 positive cancer cell mutation happens from day 120 with all cells being PDL1 positive from around day 170 and at the end of simulation at day 250 there are around 37000 cancer cell. A lower cancer killed rate of 0.01 in Figure 3c shows similar dynamics but with a further delay in time. A plateau of the cancer cell number occurs around day 130 till 150 and all cancer cells have mutated to PDL1 positive cancer cells around day 220. At the end of the simulation at day 250 the cancer cell population grows to around 25000 days. Those simulations show how important the interplay between tumour and immune cells is to control the tumour growth. If more cancer cells are being killed by immune cells that causes PDL1 positive cancer cells to emerge at an earlier time point which exhaust the immune effector cell and hence prevents elimination of cancer cells. So the PDL1 PD1 interaction counteracts the elimination of cancer cells.

Figures 4a-4f investigate the the influence of the changing cancer mutation rate on the tumour immune interaction. A low mutation rate of 0.0001 enables the immune cell to eliminate cancer cells starting from around 120 days to over 12000 eliminated cancer cell by the end of simulation on day 250 (see Figure 4a). The majority of the cancer cells stay PDL1 negative and less than 1000 cancer cells have mutated by the end of simulation. The cancer population grows to approximately 5000 cells. In comparison in Figure 4b, the mutation rate is ten times higher. This causes the cancer cell population to grow to higher numbers (around 14000 cells at day 250). The PDL1 positive mutation starts around 120 days when the elimination of cancer cells by immune cells starts. At 250 days approximately 7000 cancer cells have mutated to the PDL1 positive variant. This slows down the cancer elimination, around 7000 cancer cells have been eliminated by day 250 and the cancer cell population grows to around 14000 cells by day 250. Further increasing the mutation rate to 0.009 causes the whole cancer cell population being PDL1 positive by day 220 and a cancer cell population size of around 25000 by the end of simulation on day 250. A mutation rate of 0.05 causes all cancer cells to become PDL1 positive around day 160 and an overall population size of approximately 23000 cancer cells by the end of simulation at day 250. A mutation rate of 0.1 causes all cancer cells being PDL1 positive around day 140 and a population size of over 30000 cancer cells at the end of simulation. With the mutation rate of 1 the cancer cell population is PDL1 positive around day 110 and reaches over 30000 cells at day 250. Overall it can be seen that a high mutation rate aids cancer growth. The immune cells are less able to eliminate and control the cancer growth due to the interaction and exhaustion caused by the mutated, PDL1 positive cancer cells.

Next, the effector cell division rate and its influence on the tumour immune interaction is investigated in the Figures 5a-5f. Figures 5a and 5b show that the effector division rate of 0.0001 and 0.001 are having a similar effect on the tumour immune interaction and tumour growth.

Contrary, a higher effector cell division rate of 0.01 causes a faster growth of the cancer cell population (see Figure 5c). The cancer cell population reaches a number of around 25000 at the end of simulation on day 250 (compared to approximately 20000 cancer cells at day 250 with a rate of 0.0001 and 0.001 in Figures 5a and 5b). Additionally, all cancer cell have become PDL1 positive around day 210 and the PDL1 mutation starts around day 130 while with the lower effector cell division rates of 0.0001 and 0.001 the mutation starts around day 140 and all cancer cells are PDL1 positive by day 240 (see Figures Figures 5a and 5b). Increasing the mutation rate further to 0.05 (Figure 5d) causes a start of PDL1 mutation around day 130 similarly to the rate of 0.01 (Figure 5c) but all cancer cells have mutated to the PDL1 positive variant at and earlier time point (around day 190 in Figure 5d). The population size at the end of simulation on day 250 is around 25000 similar to the rate of 0.01. Increasing the rate further to 0.1, increases the population size to around 70000 cancer cells at the end of simulation. The cancer cells start to mutate around day 80 and by approximately day 150 all cancer cells have become PDL1 positive. Increasing the effector cell division rate to 1 has the opposite effect (Figure 5f). Now the cancer cells are being eliminated at day 11 by the immune effector cells. Around 400 cancer cells have mutated but it is not enough to exhaust the immune effector cells and stop the immune response. In summary, the analysis shows that increasing the effector cell division rate causes an increase of mutation to the PDL1 positive variant and an increase of the cancer growth until the number of effector cells is high enough to eliminate the cancer cells at an early time point. That is because more immune effector cells will eliminate more cancer cells which triggers mutation events to happen. As a consequence, more immune cell exhaustion occurs which leads to an ineffective control of the cancer growth by the immune system. If the effector cell division rate is high enough, then the cancer cells can be eliminated before the PDL1 positive variant is dominant and exhaust the immune effector cells.

Figures 6a-6e show the influence of changing the effector exhaustion rate on the tumour immune interaction. Starting with a low exhaustion rate of 0.001 in Figure 6a, cancer cells start to mutate around 130 days. Around 180 days, all cancer cells are PDL1 positive. At the end of simulation at day 250 The cancer cell population grows to a number of around 13000. A higher effector cell exhaustion rate of 0.01 in Figure 6b causes more effector cell exhaustion. Hence, less cancer cell elimination is happening and the cancer cells have less pressure to mutate to the PDL1 positive variant. This can be seen in the later time point for all cancer cells to become PDL1 positive (at approximately 210 days with the rate of 0.01 in Figure 6b versus at approximately day 180 with the rate of 0.001 in Figure 6a). At the end of simulation the cancer cell number is approximately 25000 which is almost double in comparison to the cause with an exhaustion rate of 0.001 (Figure 6a) as the immune system is less able to control the cancer growth with more exhaustion happening. Further increasing the exhaustion leads to less pressure for the cancer cells to mutate. This can be seen in the later time point for all cancer cells to become PDL1 positive with a higher effector cell exhaustion rate (200 days with the rate of 0.05 (Figure 6c), day 240 with the rates 0.1 (Figure 6d) and 1 (6e)). Additionally, the analysis shows that the immune effector cells are less able to eliminate cancer cells and slow down the cancer growth with a higher exhaustion rate. In Figure 6a a decrease in cancer cell number can be seen from day 150 to day 180 with a decrease of approximately 3000 cancer cells to 2000 cancer cells. In Figure 6b there is

no decrease but a plateau in the cancer cell number from day 130 to day 150 where the cancer cell population is around 3000 cells. With increasing the exhaustion rate the plateau is less pronounced (see Figure 6c and 6d) until there is no plateau visible in Figure 6e with the exhaustion rate of 1. In summary, this analysis shows that PDL1 mutation depends on efficient elimination of cancer cells. If less cancer cells are being eliminated due to the higher exhaustion rate, then there is less pressure for the cancer cells to mutate to the PDL1 positive variant.

Figures 7a-7f investigate the influence of the changing effector cell infiltration rate on the tumour immune interaction. In Figure 7a, a rate of 0.0001 cause a decrease in the cancer growth at 155 days from around 1500 cells to 1400 cells at day 160. Around day 150 the first cancer cell become PDL1 positive and by the end of simulation at day 250 around 1000 out of 6000 cancer cells are PDL1 positive. Increasing the effector cell infiltration rate in Figure 7b to 0.001 causes more elimination of cancer cells by the immune effector cells and hence a higher mutation rate. The cancer cells start to mutate around day 140. By the end of the simulation at day 250 around 4500 out of 7000 cancer cells are PDL1 positive. Increasing the effector cell infiltration rate further to 0.005 in Figure 7c, increases the pressure to mutate further. The cancer cells start to mutate around day 140 and by day 210 all cancer cells are PDL1 positive. Because immune effector cells become exhausted and are less likely to eliminate the PDL1 positive cancer cell, the cancer grows to higher number than in the previous two cases (22000 cancer cells at the end of simulation at day 250 in Figure 7c in comparison to 6000 and 7000 cancer cells at the end of simulation in Figures 7a and 7b). Further increasing the effector cell infiltration rate to 0.01 in Figure 7d causes and early mutation start around day 100 and by day 140 all cancer cells are PDL1 positive. The immune system is less able to control the cancer growth and by the end of simulation at day 210 the population has grown to the size of 40000 cancer cells. A further increase of the rate to 0.1 seems to reach a turning point in Figure 7e. Now there are enough immune effector cells to eliminate the cancer cells and overcome the PDL1 PD1 interaction as a counter mechanism. The cancer cell population is being eliminated around day 22. Further increasing the immune effector infiltration rate to 1 accelerate the cancer extinction. The immune system is able to erradicate the cancer cells by day 3. In summary, this analysis shows that increasing the number of immune effector cells with the infiltration rate, will trigger a higher mutation rate to the PDL1 positive variant and a higher cancer growth as exhausted immune effector cells are less able to control the cancer growth. This is happening until the effector cell infiltration rate is high enough cause a high immune effector cell number which overcomes the PDL1 PD1 exhaustion mechanism.

Figures 8a-8e show the influence of the changing effector cell moving rate on the tumour immune interaction. Starting with a low effector cell moving rate of 0.0001 in Figure 8a, it can be seen that the elimination of cancer cells by immune effector cells starts late around 200 days because the immune effector cells are moving less and need more time to reach the cancer cell population after infiltration into the tumour microenvironment. After reaching and eliminating the tumour cells, the cancer cells start mutating to the PDL1 positive variant. At the end of the simulation, cancer cell population reaches the size of around 25000 cells with as minority of around 5000 cancer cells being PDL1 positive. Increasing the moving rate to 0.02 in Figure 8c, causes the immune effector cells to reach the cancer cells at an earlier time point and the cancer cells

elimination and PDL1 mutation starts around 130 days. By day 220 all cancer cells have become PDL1 positive. At the end of the simulation around day 250 the cancer cell population has grown to a size of 30000 cells. Increasing the moving rate to 0.07 in Figure 8b, causes the immune effector cells to reach the cancer cells at an earlier time point and the cancer cells elimination and PDL1 positive mutation starts around 120 days. By day 180 all cancer cells have become PDL1 positive. At the end of the simulation around day 250 the cancer cell population has grown to a size of 30000 cells. Increasing the moving rate further to 0.1 in Figure 8d, causes the immune effector cells to reach the cancer cells at an earlier time point and the cancer cells elimination and PDL1 positive mutation starts around 110 days. By day 180 all cancer cells have become PDL1 positive. At the end of the simulation around day 250 the cancer cell population has grown to a size of 30000 cells. Increasing the moving rate even further to 1 in Figure 8e, causes the immune effector cells to reach the cancer cells at an earlier time point and by day 140 all cancer cells have become PDL1 positive. At the end of the simulation around day 250 the cancer cell population has grown to a size of 200000 cells. In summary, the analysis shows that increasing the effector moving rate causes an earlier cancer cell elimination but also an earlier start of mutation to the PDL1 positive variant and a faster growth as the immune system is less able to control the cancer growth with exhausted immune effector cells.

Figures 9a-9e show the influence of the changing suppressor cell infiltration rate on the tumour immune interaction. Figures 9a and 9b with the suppressor cell infiltration rate of 0.0001 and 0.0005 show similar dynamics with the cancer cells starting to mutate around day 140 and by approximately 210 days all cancer cells in the population are PDL1 positive. At the end of the simulation the cancer cell population grows to a size of around 21000 and 25000 cells. Increasing the suppressor cell infiltration rate to 0.001 in Figure 9c shows a similar start of PDL1 mutation and population size at the end of simulation but it takes until day 240 for all cancer cells to become PDL1 positive. This is because with the higher suppressor cell infiltration rate, more immune suppressor cells are in the tumour microenvironment. The interation of those cells with the immune effector cells causes effector cell exhaustion. Hence, less elimination of cancer cells by immune effector cells is possible and hence there is less pressure to mutate for the cancer cells. This can be seen in the number of exhausted immune effector cells at the end of the simulation. In Figure 9a with the rate of 0.0001 and Figure 9b with the rate 0.0005 around 2500 immune effector cells are exhausted at the end of the simulation at day 250, while with the rate of 0.001 in Figure 9c there are around 3500 exhausted immune effector cells at the end of simulation at day 250. This trend can be observed when increasing the suppressor cells infiltration rate further. With a rate of 0.01 in Figure 9d at the end of the simulation 5000 immune effector cells are exhausted and out of 400000 cancer cells around 35000 are PDL1 positive. With a higher rate of 0.1 in Figure 9e there are around 10000 exhausted immune effector cells at the end of the simulation. The cancer cell population grows to over 80000 cells with around 18000 PDL1 positive cancer cells. In summary, the trend can be observed that with at higher suppressor infiltration rate, the cancer cells have less pressure to mutate. The immune effector cells become exhausted by the interaction with the high number of immune suppressor cells instead of becoming exhausted by interaction with the PDL1 positive cancer cells. Due to the exhaustion the immune effector cells are less able to eliminate the

cancer cells and hence cause less pressure to mutate. With the increasing suppressor cell infiltration rate and the immune effector cells exhaustion, the immune cells are less able to control the cancer growth and the cancer cell population size at the end of the simulation is larger with a higher suppressor cell infiltration rate.

Similar dynamics can be seen with increasing the suppressor cell moving rate investigated in Figures 10a-10d. With a low rate of 0.001 in Figure 10a, the whole cancer cell population become PDL1 positive around day 210. Increasing the suppressor cell moving rate to 0.07 in Figure 10b and 0.1 in Figure 10c, causes the whole cancer cell population to be PDL1 positive at day 230. The suppressor cell moving rate of 1 in Figure 10d shifts the time point for all cancer cells to be PDL1 positive beyond the end time of the simulation at day 250. This phenomena is explained by the immune suppressor cells being more mobile and hence reaching more immune effector cells to exhaust. With an increasing number of exhausted immune effector cells there is less cancer cell elimination and less pressure to mutate to the PDL1 positive variant.

1.2 Comparing treatment options

In this section, plots of different treatment options from the results section in the main manuscript are displayed next to each other for an easier comparison. Additionally, a combination treatment with radiotherapy with a dose of 2.5 Gy/day for 5 days/week for 6 weeks starting at day 100 and weekly 210 mg/m2 DNA damage response inhibitor starting at day 150 is shown.

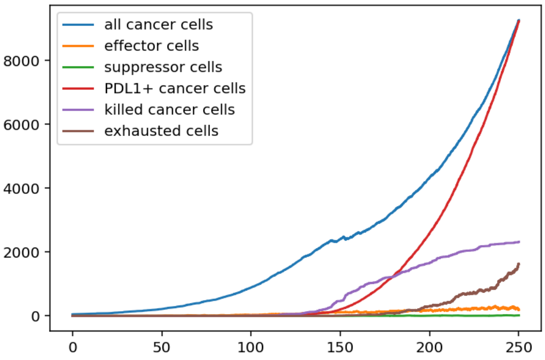

(a) Cancer death rate = 0.001

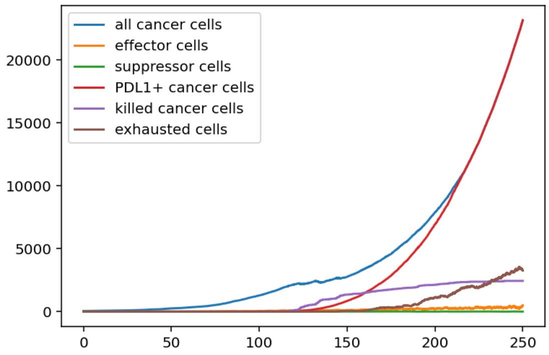

(b) Cancer death rate = 0.0001

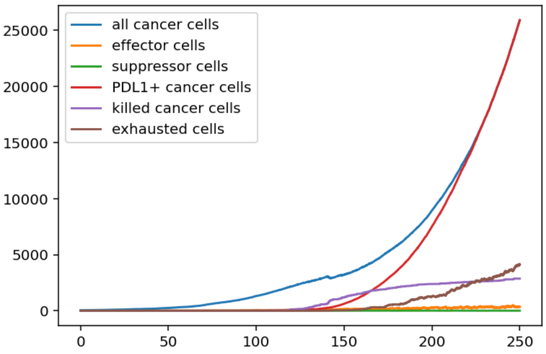

(c) Cancer death rate = 0.00001

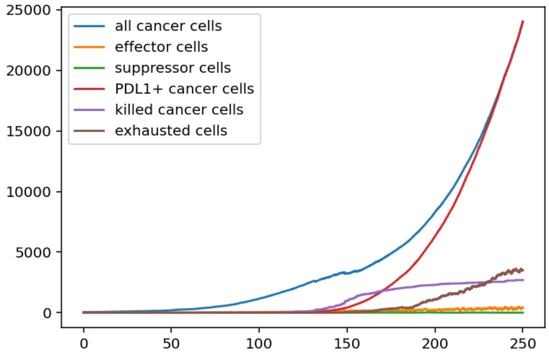

(d) Cancer death rate = 0.000005

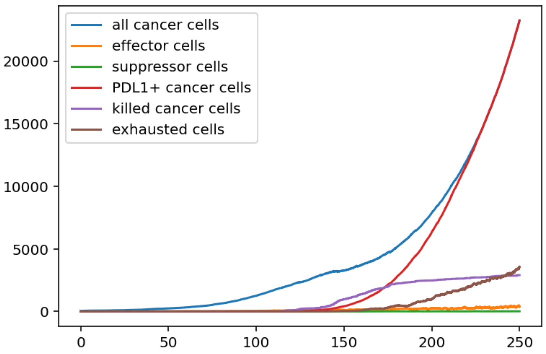

(e) Cancer death rate = 0.0000001

Figure 1: Figures 1a-1e show the influence of the changing cancer death rate on the tumour immune interaction.

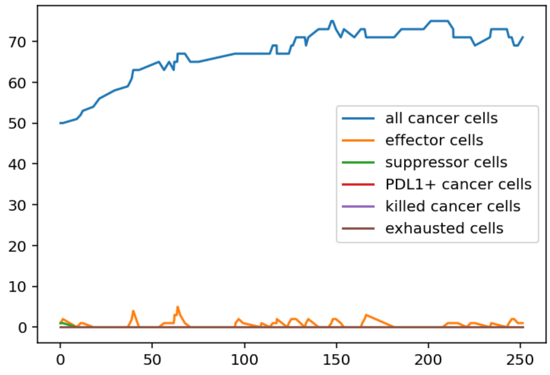

(a) Cancer division rate = 0.0001

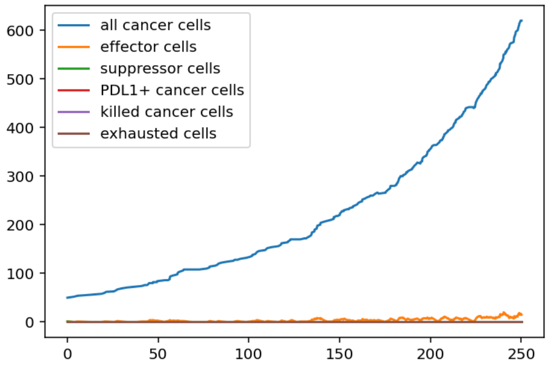

(b) Cancer division rate = 0.001

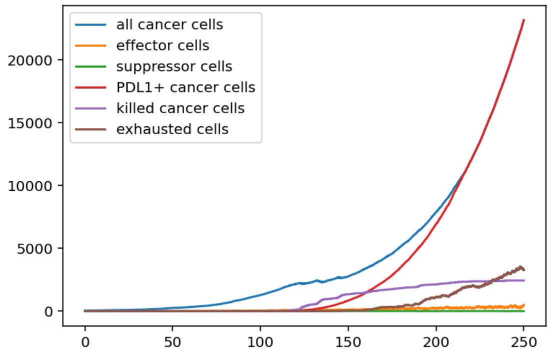

(c) Cancer division rate = 0.01

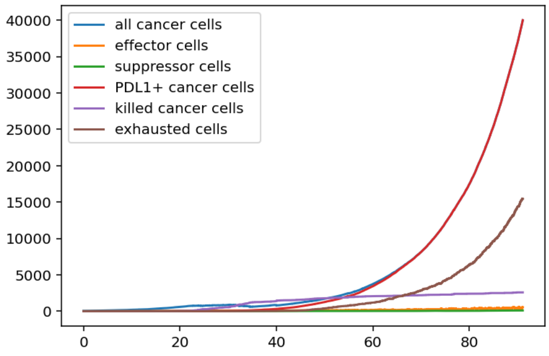

(d) Cancer division rate = 0.05

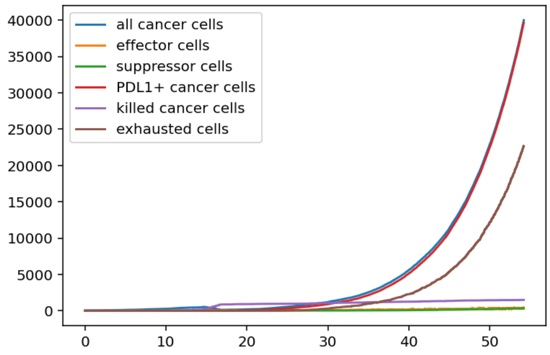

(e) Cancer division rate = 0.1

Figure 2: Figures 2a-2e show the influence of the changing cancer divison rate on the tumour immune interaction.

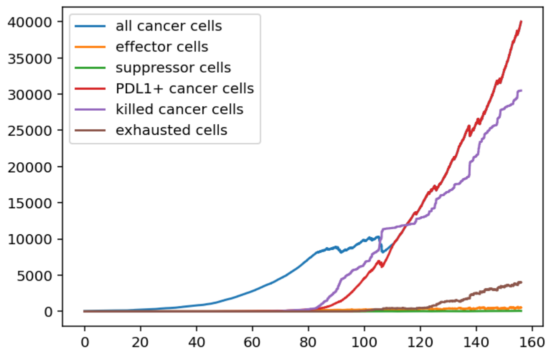

(a) Cancer killed rate = 1

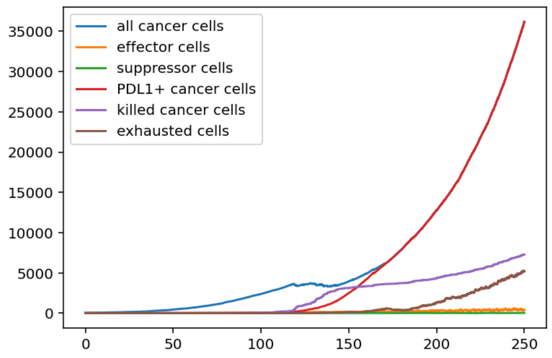

(b) Cancer killed rate = 0.1

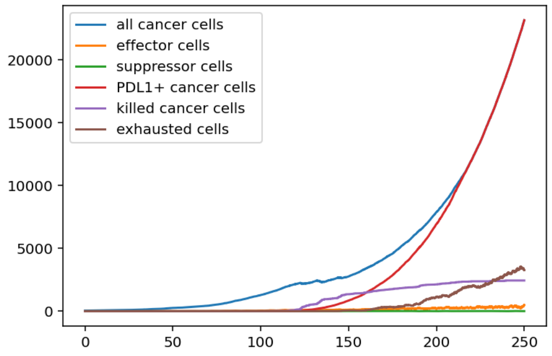

(c) Cancer killed rate = 0.01

Figure 3: Figures 3a-3c show the influence of the changing cancer killing rate on the tumour immune interaction.

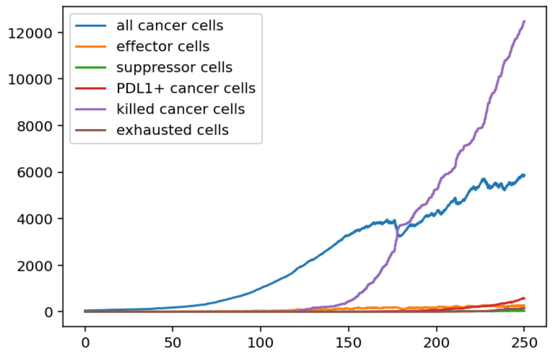

(a) Cancer mutation rate = 0.0001

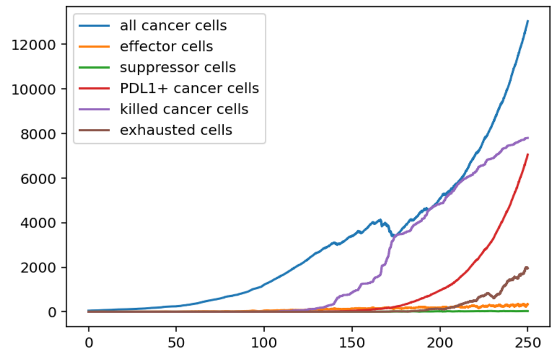

(b) Cancer mutation rate = 0.001

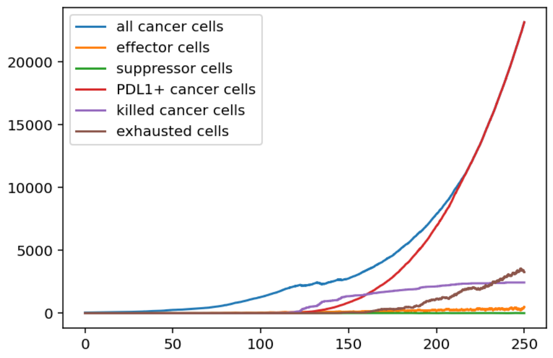

(c) Cancer mutation rate = 0.009

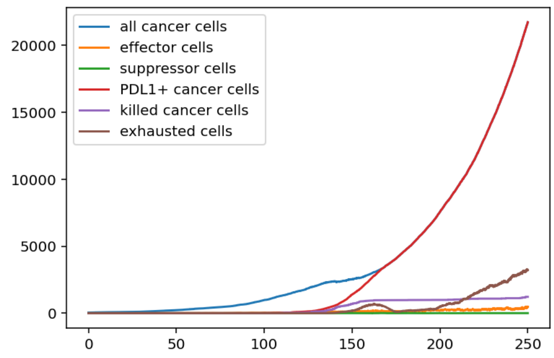

(d) Cancer mutation rate = 0.05

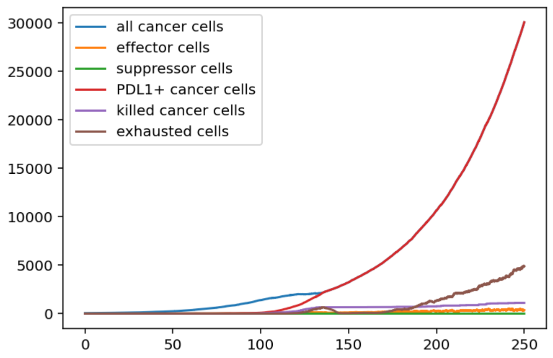

(e) Cancer mutation rate = 0.1

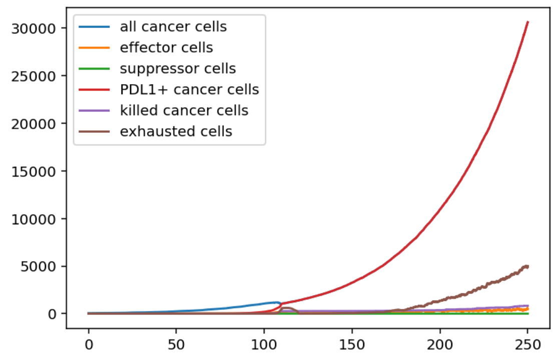

(f) Cancer mutation rate = 1

Figure 4: Figures 4a-4f show the influence of the changing cancer mutation rate on the tumour immune interaction.

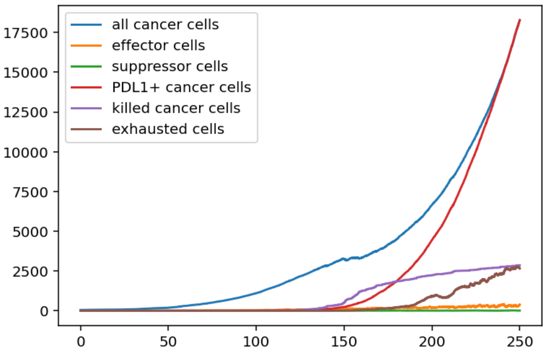

(a) Effector cell division rate = 0.0001

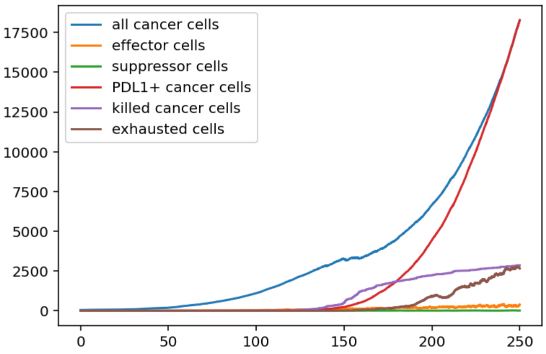

(b) Effector cell division rate = 0.001

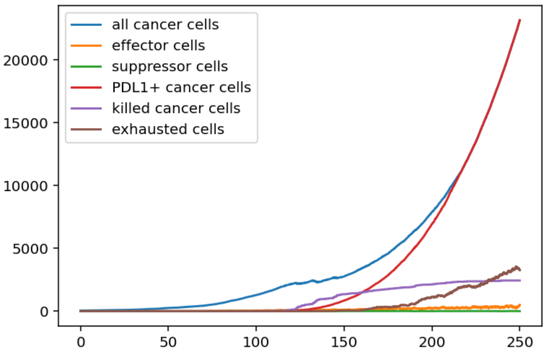

(c) Effector cell division rate = 0.01

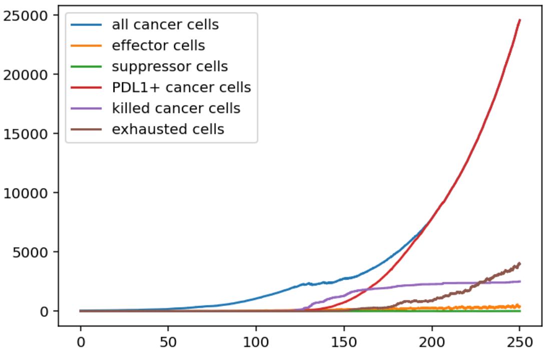

(d) Effector cell division rate = 0.05

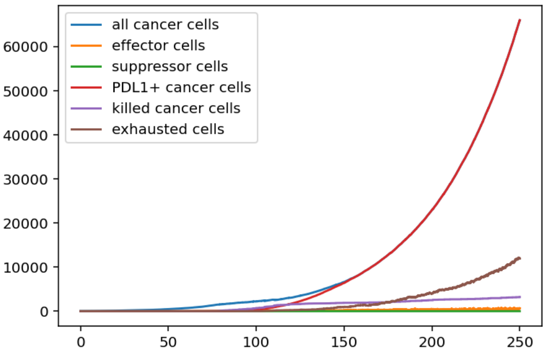

(e) Effector cell division rate = 0.1

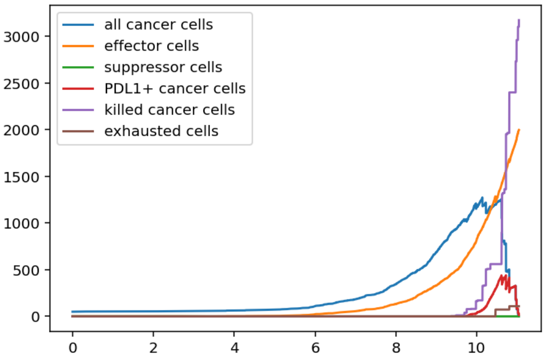

(f) Effector cell division rate = 1

Figure 5: Figures 5a-5f show the influence of the changing effector cell division rate on the tumour immune interaction.

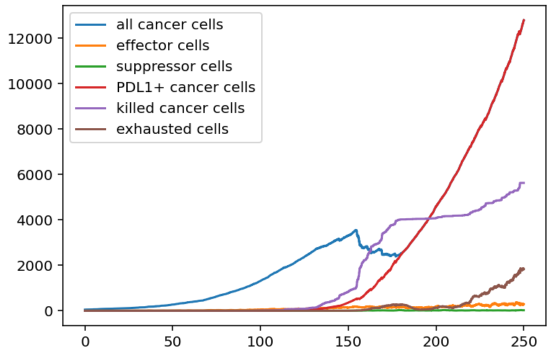

(a) Effector cell exhaustion rate = 0.001

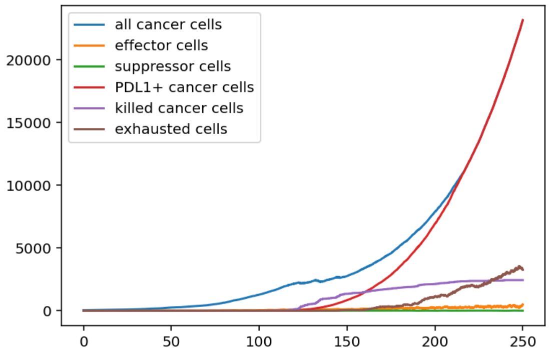

(b) Effector cell exhaustion rate = 0.01

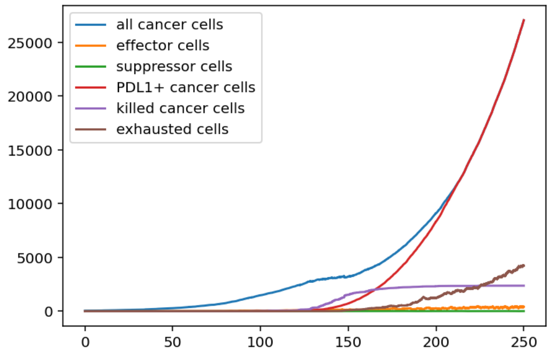

(c) Effector cell exhaustion rate = 0.05

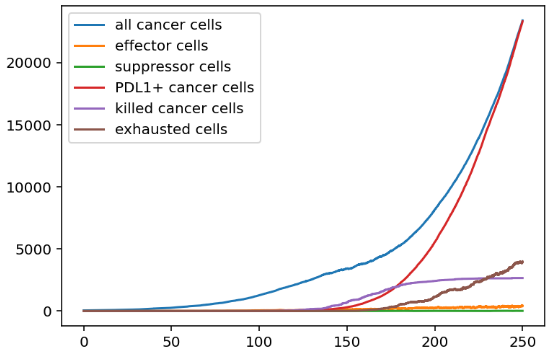

(d) Effector cell exhaustion rate = 0.1

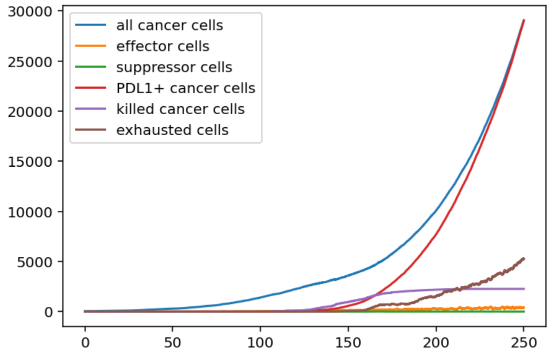

(e) Effector cell exhaustion rate = 1

Figure 6: Figures 6a-6e show the influence of the changing effector cell exhaustion rate on the tumour immune interaction.

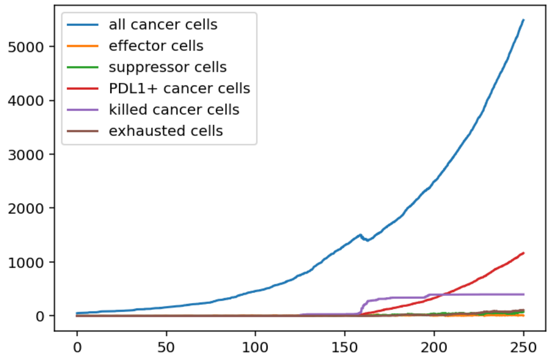

(a) Effector cell infiltration rate = 0.0001

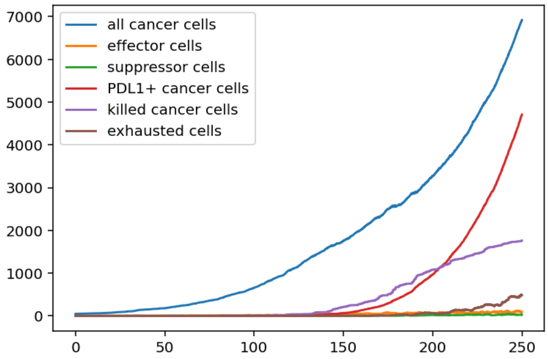

(b) Effector cell infiltration rate = 0.001

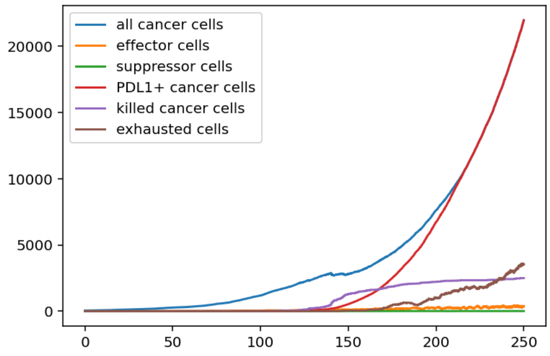

(c) Effector cell infiltration rate = 0.005

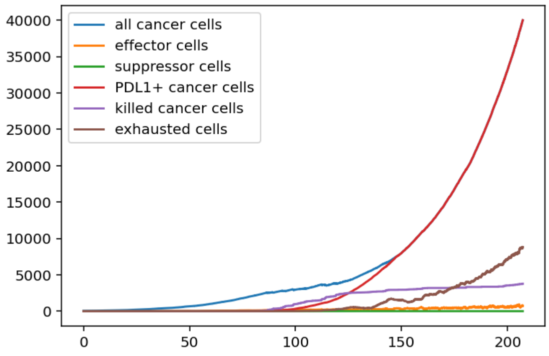

(d) Effector cell infiltration rate = 0.01

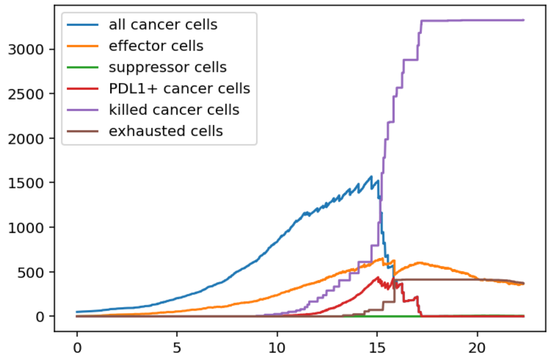

(e) Effector cell infiltration rate = 0.1

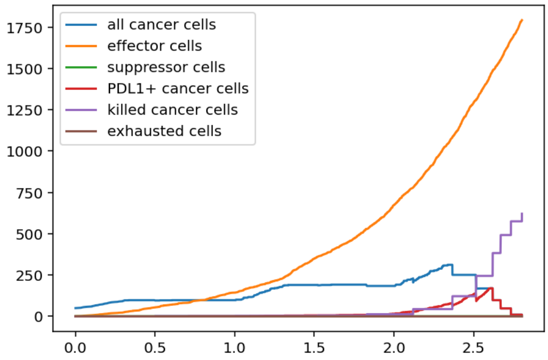

(f) Effector cell infiltration rate = 1

Figure 7: Figures 7a-7f show the influence of the changing effector cell infiltration rate on the tumour immune interaction.

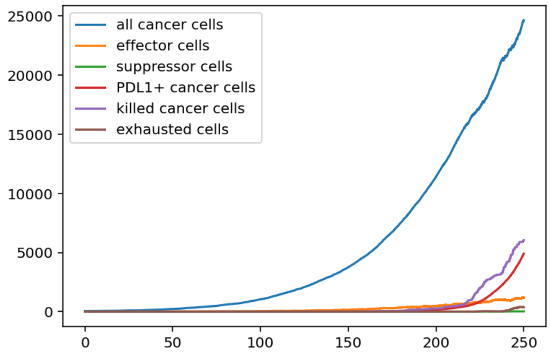

(a) Effector cell moving rate = 0.001

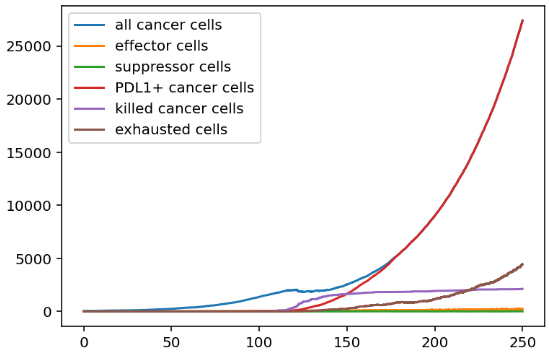

(b) Effector cell moving rate = 0.07

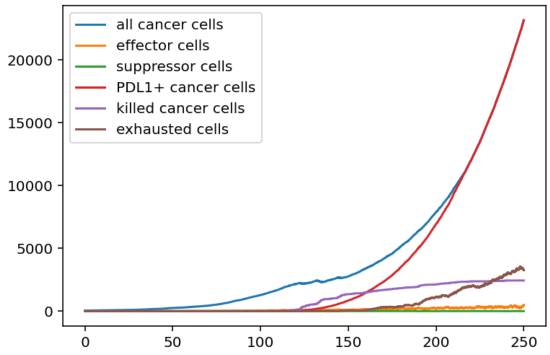

(c) Effector cell moving rate = 0.02

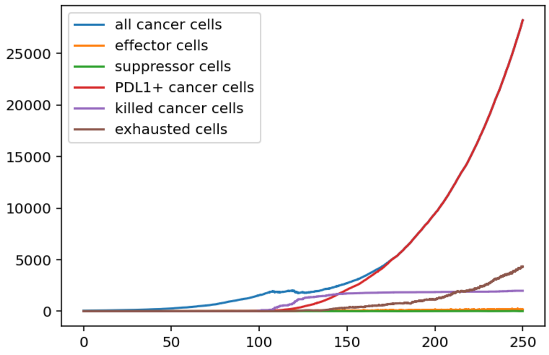

(d) Effector cell moving rate = 0.1

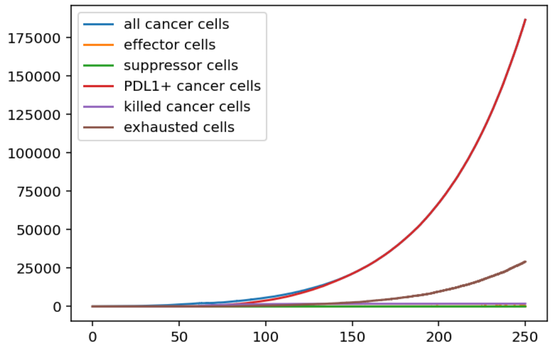

(e) Effector cell moving rate = 1

Figure 8: Figures 8a-8e show the influence of the changing effector cell moving rate on the tumour immune interaction.

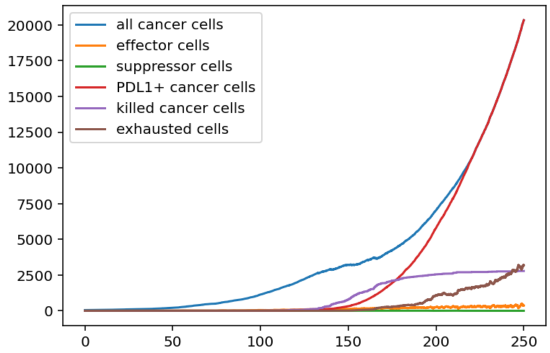

(a) Suppressor cell infiltration rate = 0.0001

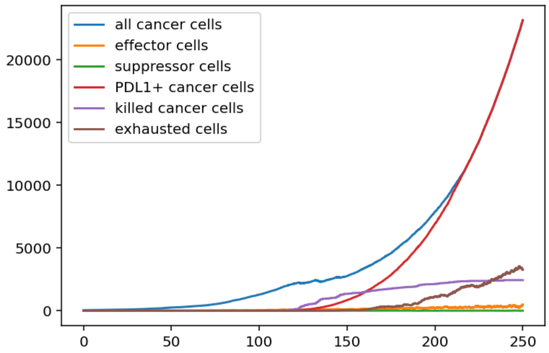

(b) Suppressor cell infiltration rate = 0.0005

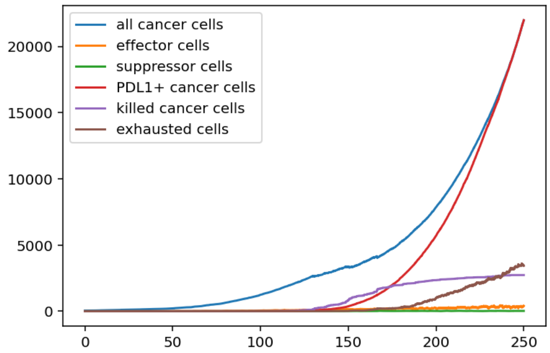

(c) Suppressor cell infiltration rate = 0.001

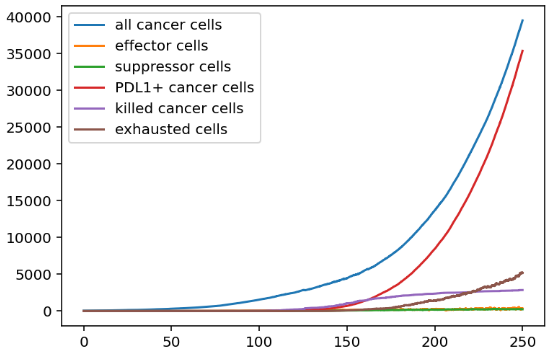

(d) Suppressor cell infiltration rate = 0.01

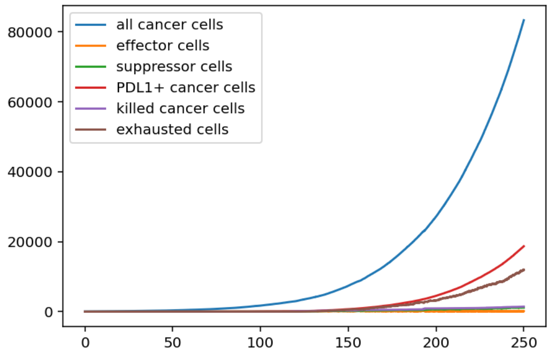

(e) Suppressor cell infiltration rate = 0.1

Figure 9: Figures 9a-9e show the influence of the changing suppressor cell infiltration rate on the tumour immune interaction.

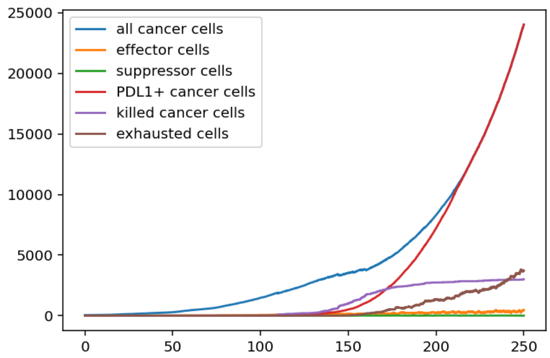

(a) Suppressor cell moving rate = 0.001

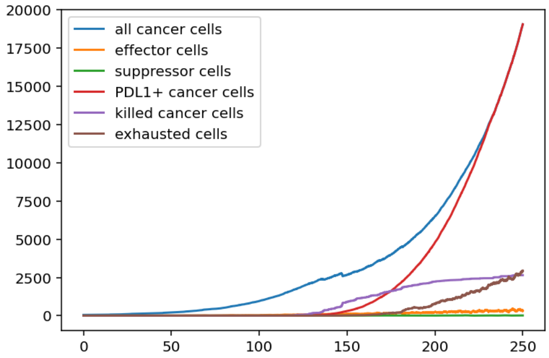

(b) Suppressor cell moving rate = 0.07

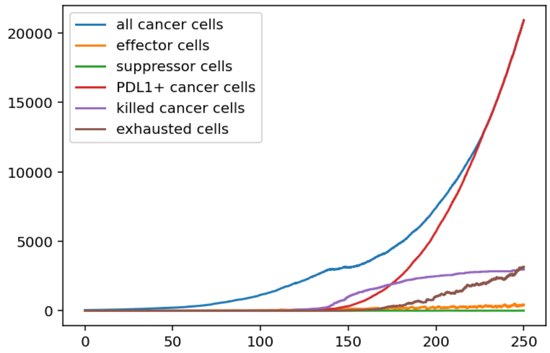

(c) Suppressor cell moving rate = 0.1

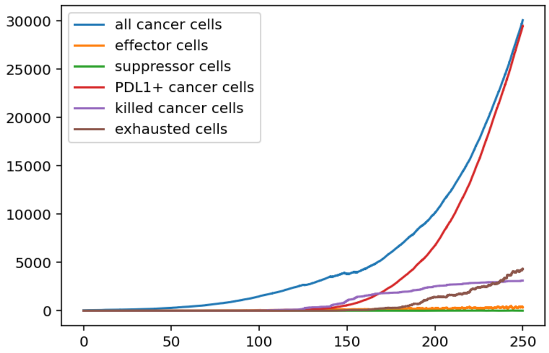

(d) Suppressor cell moving rate = 1

Figure 10: Figures 10a-10d show the influence of the changing suppressor cell moving rate on the tumour immune interaction.

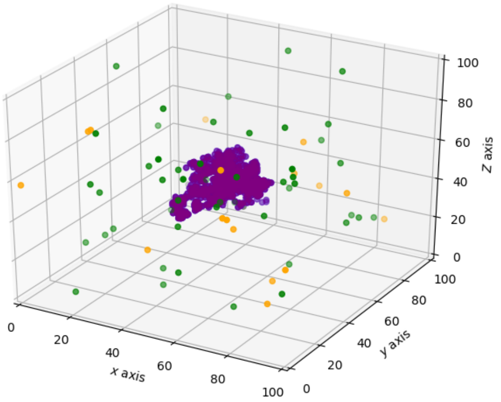

(a) TME of the DNA damage response inhibitor treatment at the end of simulation (day 300)

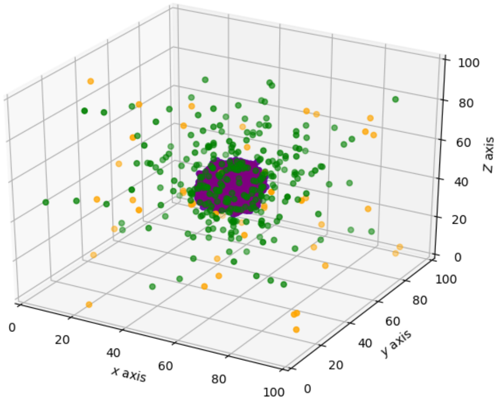

(b) TME of the PD1 antibody treatment starting at day 100 at the end of simulation (day 300)

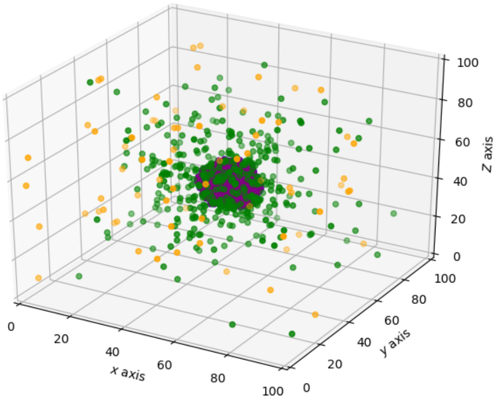

(c) TME of the PD1 antibody treatment starting at day 200 at the end of simulation (day 300)

Figure 11: Figures 11a – 11c show the the tumour microenvironment at the end of simulation (day 300) of the DNA damage response inhibitor treatment, the PD1 antibody treatment starting at day 100 and 200 with the PDL1 positive cancer cells in purple, the infiltrated immune effector cells in green and the suppressor cells in orange.

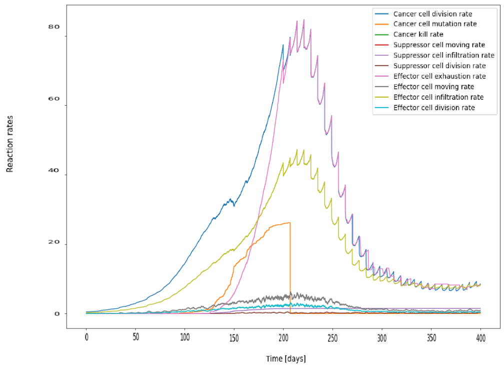

(a) Reaction rates of the DNA damage inhibitor treatment

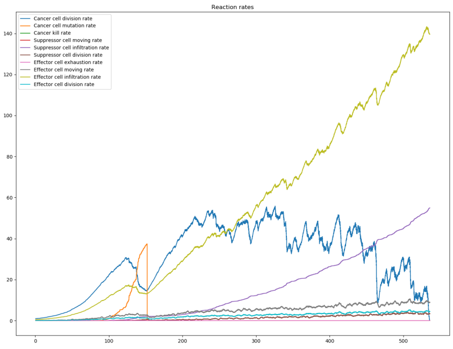

(b) Reaction rates of the PD1 antibody treatment starting at day 100

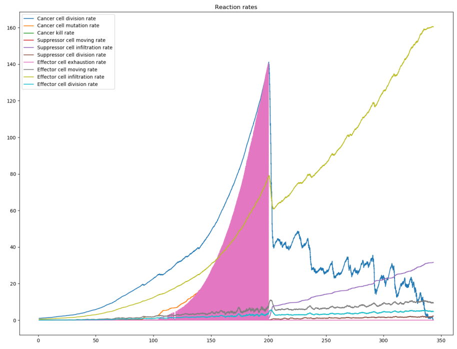

(c) Reaction rates of the PD1 antibody treatment starting at day 200. The effector cell exhaustion rate (pink line) oscillates between 0 and a value bigger than zero. The rate is 0 if no suppressor cell or PDL1 positive cancer cell is next to effector cell. As the simulation goes on and more effector cells are close to PDL1 positive cancer cells and more suppressor cells are next to the effector cells. The rate increases until the PD1 receptors of the are fully occupied by the PD1 antibody. This causes the exhaustion rate being 0 after 200 days.

Figure 12: Figures 12a – 12c show the reaction rates of the DNA damage response inhibitor treatment, the PD1 antibody treatment starting at day 100 and 200.

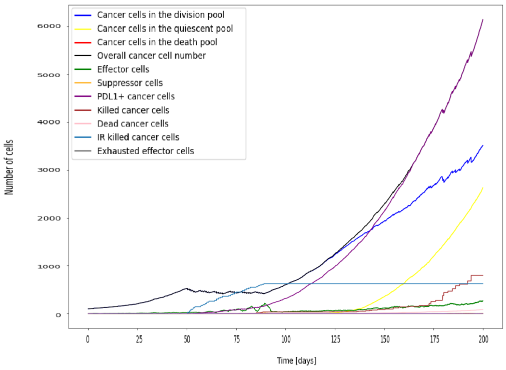

(a) Cell count over time with combination of 2mg/kg Q3W PD1 antibody treatment starting at day 20 with radiation of 2.5 Gy 5 days a week starting at day 50 for 6 weeks

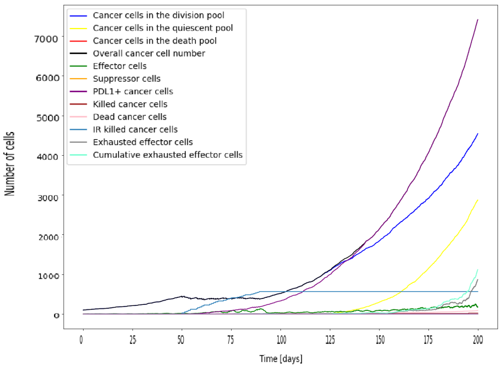

(b) Cell count over time with a radiotherapy of 2.5 Gy 5 days a week starting at day 50 for 6 weeks

Figure 13: Figures 13a – 13b the effect of the combination of PD1 antibody and radiation and radiation alone

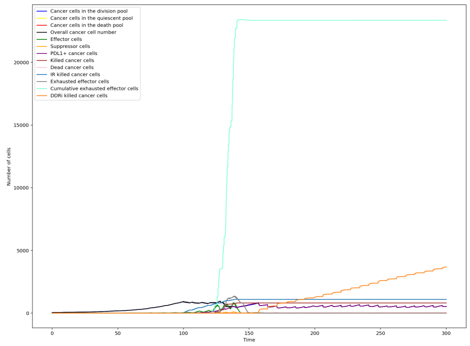

(a) Cell count over time with a combination treatment with radiation and DNA damage response inhibitor

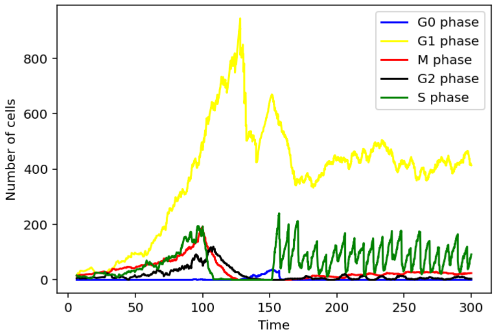

(b) Cell cycle phase distribution over time with a combination treatment with radiation and DNA damage response inhibitor

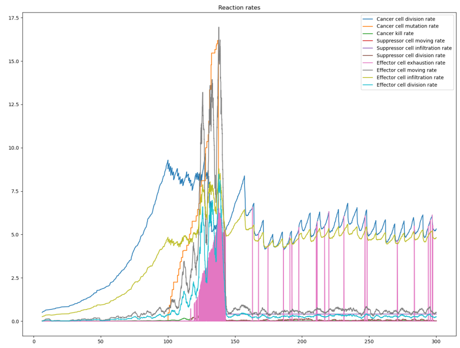

(c) Reaction rates of the combination treatment with radiation and DNA damage response inhibitor

Figure 14: Figures 14a-14c show the effect of radiotherapy with a dose of 2.5 Gy/day for 5 days/week for 6 weeks starting at day 100 and weekly 210 mg/m2 DNA damage response inhibitor starting at day 150
